# Advances in Multidimensional Molecular Mechanisms of Epilepsy and Precision Therapy Strategies

**DOI:** 10.1002/cns.71224

**Published:** 2026-11-04

**Authors:** Yimin Han, Jingxuan Xu, Yuntian Shen, Jitai Zhang, Xia Li, Xinlei Yao, Lei Qi, Hualin Sun

**Affiliations:** ^1^ Jiangsu Key Laboratory of Tissue Engineering and Neuroregeneration, Co‐Innovation Center of Neuroregeneration Nantong University Nantong Jiangsu People's Republic of China; ^2^ Department of Emergency Medicine Affiliated Hospital of Nantong University Nantong Jiangsu People's Republic of China

**Keywords:** epilepsy, ion channels, neuroinflammation, pharmacoresistance

## Abstract

**Introduction:**

Epilepsy is a complex neurological disorder characterized by recurrent seizures. A substantial proportion of patients develop drug‐resistant epilepsy (DRE), highlighting the limitations of current therapies and the need to view epilepsy beyond the traditional “channelopathy” hypothesis as a multifactorial network disorder.

**Method:**

This narrative review synthesizes current evidence on epilepsy pathogenesis, mechanisms of drug resistance, and emerging therapeutic strategies. Relevant studies were identified through literature searches of PubMed and Web of Science, with emphasis on publications from 2018 to 2025 and landmark studies that have shaped current understanding of epileptogenesis, drug‐resistant epilepsy, and precision therapy.

**Results:**

The pathogenesis of epilepsy converges genetic, neuroinflammatory, and cellular homeostasis pathways. Key drivers include ion channel mutations, glia‐mediated neuroinflammation, and dysregulated processes like autophagy and ferroptosis. These contribute to drug resistance and comorbidities, driving a therapeutic shift toward precision and network‐modulation strategies, such as gene therapy, targeted pathway modulation, and neuromodulation, with the ultimate goal of achieving disease modification rather than seizure suppression alone.

**Conclusions:**

Integrating multi‐omics insights to construct a holistic precision medicine framework, spanning from molecular diagnosis to tailored network therapy, is fundamental for advancing epilepsy treatment. Future efforts must focus on translating this integrated understanding into effective clinical strategies to overcome therapeutic resistance.

## Graphical abstract

Epilepsy arises from a multidimensional network imbalance, integrating genetic, inflammatory, and metabolic disruptions. This complexity drives neuronal hyperexcitability and underpins therapeutic challenges. Our review highlights how emerging precision strategies, from targeted molecular therapies to neuromodulation, offer a transformative path toward personalized disease management.

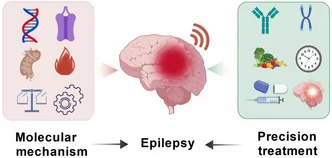

## 1 Introduction

Epilepsy is a chronic neurological disorder affecting approximately 50 million people worldwide. It is characterized by recurrent seizures resulting from abnormally hypersynchronous neuronal discharges, which severely impact patients' quality of life and impose significant neurofunctional burdens [1]. Although clinically and etiologically heterogeneous, the core pathophysiology is widely recognized as an imbalance between excitation and inhibition in the central nervous system. Recent advances in molecular genetics, epigenetics, and neuroimmunology have expanded our understanding of epilepsy pathogenesis beyond the traditional “channelopathy” concept. It is now viewed as a multidimensional network disorder involving neuroinflammation, metabolic disturbances, disrupted cellular homeostasis, and neural‐network remodeling [2, 3]. About one‐third of patients develop DRE, underscoring the limitations of current therapies and highlighting the complex polygenic and multipathway interactions underlying the disease.

At the molecular level, epilepsy pathogenesis encompasses genetic variations, ion‐channel dysfunction, neurotransmitter system imbalances, dysregulation of cellular processes (autophagy, apoptosis, ferroptosis), epigenetic modifications, and metabolic‐mitochondrial abnormalities. Over 40 ion‐channel genes have been linked to epilepsy, with mutations in genes such as SCN1A, KCNQ2, and KCNT1 directly affecting neuronal excitability and network synchronization [4, 5]. Non‐ion‐channel genes (e.g., PCDH19, HCFC1, SLC1A2) further contribute through diverse mechanisms including disrupted cell adhesion, transcriptional regulation, and synaptic glutamate clearance [6, 7]. Neuroinflammation, driven by activated glia and peripheral immune infiltration, has emerged as a key pathogenic factor that enhances neuronal excitability and perpetuates seizure activity [8]. Epigenetic mechanisms—DNA methylation, histone modifications, non‐coding RNAs—also critically regulate epilepsy‐related gene expression and network stability [3, 9].

Despite continuous innovation in anti‐seizure medications (ASMs), drug resistance remains a major challenge, involving alterations in drug targets, overexpression of efflux transporters at the blood–brain barrier, and large‐scale epileptic network remodeling [10, 11, 12]. Future therapeutic strategies are increasingly shifting toward precision and multi‐target interventions, including targeted therapies for specific genetic defects, modulation of key signaling pathways, epigenetic approaches, neuromodulation, and disease‐modifying strategies targeting epileptogenesis. Unlike many existing reviews that focus on individual pathogenic mechanisms, this review considers epilepsy as a multidimensional network disorder and integrates evidence across seven interconnected mechanistic domains: genetic and ion channel abnormalities, neurotransmitter imbalance, neuroinflammation, cellular homeostasis disruption, epigenetic dysregulation, metabolic–mitochondrial dysfunction, and neural network/neurovascular remodeling. Particular emphasis is placed on mechanistic convergence and pathological crosstalk, as well as their implications for emerging precision therapeutic strategies. By linking molecular mechanisms to translational opportunities, this review aims to provide an integrated framework for understanding epilepsy pathogenesis and future therapeutic development. What distinguishes this review from previous overviews is its emphasis on mechanistic convergence across genetic, inflammatory, metabolic, epigenetic, and network‐level processes, together with a translational framework linking these interconnected mechanisms to emerging precision therapeutic strategies and disease‐modifying interventions.

## 2 Molecular Mechanisms of Epilepsy

Epilepsy is a complex neurological disorder characterized by recurrent, unprovoked seizures, arising from aberrant electrical activity in the brain. Its pathogenesis is not attributed to a single cause but rather involves a multifaceted interplay of various molecular and cellular mechanisms. Current research has shifted from a purely neuron‐centric view to a more integrated approach, recognizing that the condition emerges from the complex interplay of genetic predisposition, ion channel dysfunction, imbalances in neurotransmitter systems, and neuroinflammatory processes. Furthermore, disruptions in cellular homeostasis, epigenetic regulation, metabolic pathways, and large‐scale neural networks are increasingly understood as critical contributors to epileptogenesis and the progression of the disease (Figure 1). This section delves into these core mechanisms, beginning with the foundational roles of genetic factors and ion channel abnormalities to provide a comprehensive overview of the pathophysiological landscape of epilepsy.

**FIGURE 1:**
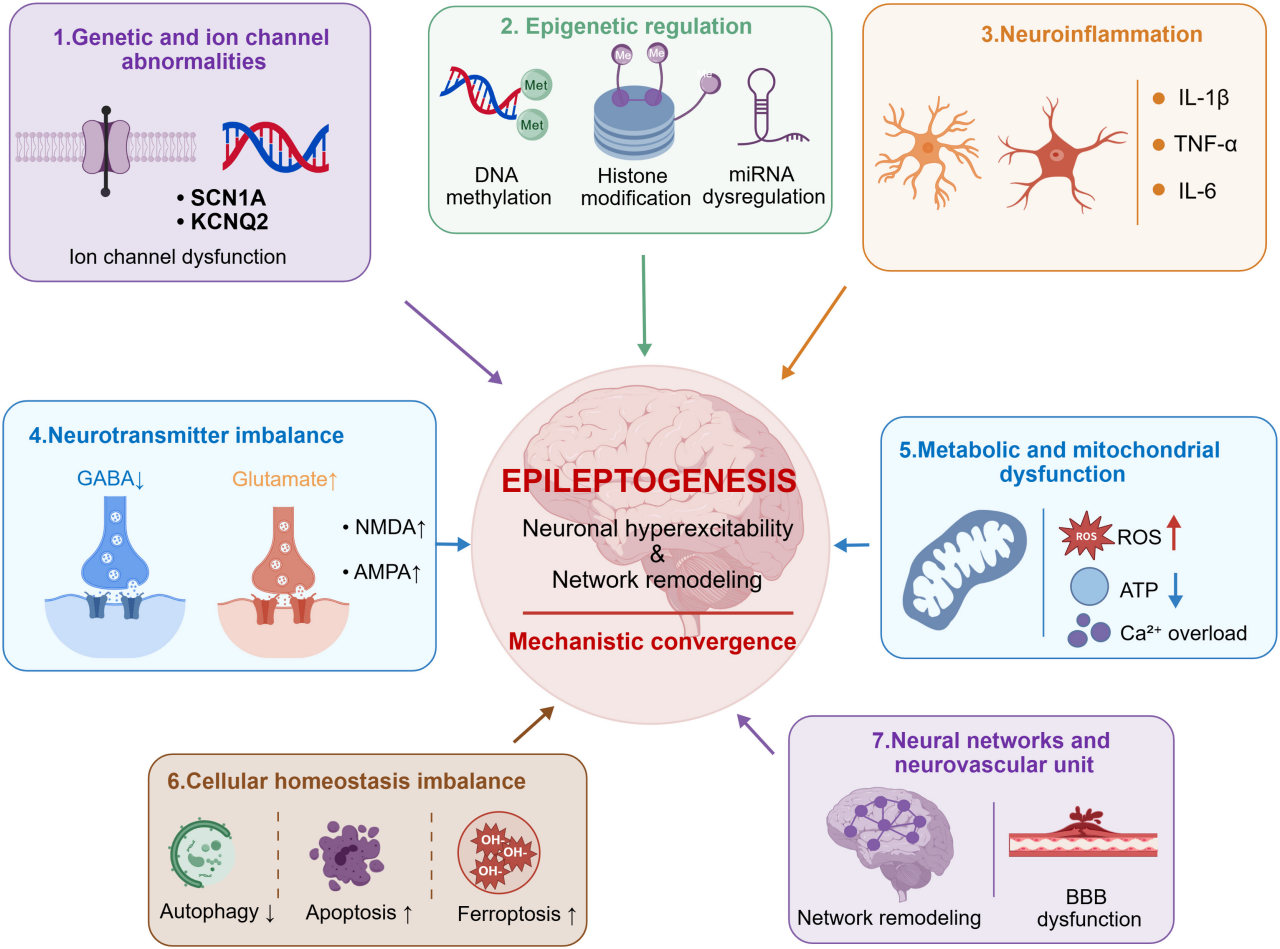
Integrated mechanisms of epileptogenesis. The figure summarizes the major interconnected pathogenic mechanisms involved in epilepsy, including genetic and ion channel abnormalities, neurotransmitter imbalance, neuroinflammation, epigenetic dysregulation, metabolic and mitochondrial dysfunction, cellular homeostasis disruption, and neural network/neurovascular abnormalities. These mechanisms interact extensively and converge on neuronal hyperexcitability and network remodeling, ultimately driving epileptogenesis, disease progression, and treatment resistance. Created with BioGDP.com [13].

### 2.1 Genetic and Ion Channel Abnormalities

Epilepsy exhibits significant genetic heterogeneity, and its genetic basis encompasses various forms, such as single‐gene mutations and copy number variations (CNVs). These genetic variations directly participate in the pathogenesis of epilepsy by influencing neuronal excitability, synaptic function, and brain development. Among these, ion channel dysfunction represents a core mechanism.

#### 2.1.1 Direct Effects of Genetic Factors

Epilepsy exhibits significant genetic heterogeneity, with approximately 30% of cases linked to single‐gene mutations, especially in childhood‐onset forms [14]. Advances in next‐generation sequencing have identified numerous epilepsy‐associated genes involved in neuronal excitability, synaptic transmission, and brain development. Representative examples include RYR3, which regulates intracellular calcium signaling, and GABRB3, which encodes a subunit of the GABAA receptor; pathogenic variants in these genes have been associated with focal epilepsy, developmental and epileptic encephalopathies, and neurodevelopmental abnormalities [15, 16]. In addition to single‐gene mutations, copy number variations (CNVs) involving loci such as CNTNAP2 and CHRNA7 further contribute to epilepsy susceptibility and phenotypic diversity [17]. Collectively, these findings highlight the diverse genetic architecture underlying epileptogenesis.

#### 2.1.2 Core Mechanism of Ion Channel Dysfunction

Regarding sodium channel abnormalities, the SCN8A gene encodes the voltage‐gated sodium channel Nav1.6. Missense mutations in this gene are closely associated with Early Infantile Epileptic Encephalopathy type 13, also known as SCN8A‐related epilepsy. Affected patients often present with multiple DRE types, developmental delays, motor impairments, and a high risk of Sudden Unexpected Death in Epilepsy (SUDEP). Studies using neurons differentiated from induced pluripotent stem cells have further revealed that SCN8A mutations lead to increased persistent or resurgent sodium currents, delayed action potential repolarization, and shortened axon initial segments. These alterations collectively enhance neuronal excitability and network hyperexcitability [18]. Notably, different mutation sites such as p.R1872L, p.V1592L, and p.N1759S contribute to pathogenesis through distinct electrophysiological mechanisms, providing a theoretical basis for precise targeted interventions [18]. Research on the pathological mechanisms of sodium channels continues to drive the advancement of epilepsy treatment strategies.

In the context of potassium channel abnormalities, the KCNB1 gene encodes the voltage‐gated potassium channel Kv2.1. Mutations in this gene can lead to developmental and epileptic encephalopathy (DEE) or developmental encephalopathy. Studies indicate that missense mutations are often associated with more frequent and severe seizures, whereas truncating mutations generally result in milder phenotypes. Long‐term follow‐up data reveal that patients with KCNB1‐related encephalopathy commonly exhibit poor cognitive outcomes and frequent autistic features. Moreover, the epilepsy course is highly heterogeneous [19]. These findings highlight the important role of potassium channel dysfunction in shaping seizure severity, neurodevelopmental outcomes, and disease heterogeneity [19].

Regarding abnormalities in calcium channels, the CACNA1A gene encodes P/Q‐type calcium channels. Mutations in this gene are linked to childhood absence epilepsy, ataxia, and dystonia. In adult mice, induced deletion of Cacna1a recapitulates the phenotypes of congenital deletion. However, the epileptogenic mechanism does not rely on enhanced T‐type calcium currents. Instead, it promotes seizures by impairing burst firing in thalamic reticular nucleus neurons [20]. The diversity and functional distribution of calcium channels thus provide a deeper perspective for understanding epileptogenesis mechanisms.

#### 2.1.3 Interplay Between Genetic Factors and Ion Channel Dysregulation

The pathogenesis and progression of epilepsy involve multilevel and complex interactions between genetic factors and ion channel abnormalities. Key signaling pathways, such as the PI3K‐AKT‐mammalian target of rapamycin (mTOR) pathway, regulate neuronal excitability by integrating cellular growth and metabolic processes. Genetic dysregulation of these pathways—for instance, mutations in PTEN or TSC1/2 genes—can lead to pathway hyperactivation and contribute to epileptogenesis [14]. Epigenetic mechanisms, particularly DNA methylation, influence epilepsy susceptibility by modulating the expression of ion channel genes such as GABRB1, KCNA2, and HCN1. The transcriptional repressor complex RE1‐silencing transcription factor/neuron‐restrictive silencer factor (REST/NRSF) plays a crucial role in linking epigenetic regulation to ion channel function [3, 21]. Recent studies have also revealed the role of non‐coding region variants in epilepsy. For example, DNA repeat expansions in intronic regions of genes such as SAMD12—specifically, TTTCA and TTTTA repeats—have been identified as causes of benign adult familial myoclonic epilepsy (BAFME). This finding expands our understanding of the genetic basis of epilepsy, demonstrating that non‐coding variations can also lead to neuronal dysfunction [22]. In summary, the etiological network of epilepsy spans multidimensional mechanisms, including signaling pathways, epigenetic regulation, and the non‐coding genome. Future research should further integrate these aspects to advance the development of precision therapy strategies.

#### 2.1.4 Mutations in Non‐Ion Channel Genes

Mutations in non‐ion channel genes also contribute substantially to epilepsy pathogenesis through mechanisms involving neurodevelopment, synaptic regulation, and network formation. Heterozygous mutations in PCDH19 cause PCDH19 clustering epilepsy through a unique cellular interference mechanism, whereby the coexistence of wild‐type and mutant cells disrupts cortical development and neuronal network organization [6, 23]. In addition, SLC1A2 encodes a major glutamate transporter responsible for synaptic glutamate clearance, and pathogenic variants impair excitatory neurotransmission homeostasis, contributing to developmental and epileptic encephalopathies [7]. Another well‐characterized example is Dup15q syndrome, in which UBE3A overexpression promotes neuronal hyperexcitability through altered sodium channel function and network activity [24]. Additional epilepsy‐associated genes, including HCFC1, YWHAG, and ATP2B1, further expand the genetic landscape of epilepsy and highlight the contribution of diverse pathways involving transcriptional regulation, intracellular signaling, and calcium homeostasis to disease susceptibility and phenotypic variability [25, 26, 27]. Collectively, these findings demonstrate that epilepsy‐associated genes influence neuronal excitability, synaptic transmission, brain development, and network formation through multiple complementary mechanisms. Beyond their direct molecular effects, many of these genetic alterations interact with neurotransmitter systems, epigenetic regulation, and intracellular signaling pathways, illustrating how genetic susceptibility is integrated into the broader epileptogenic network.

### 2.2 Imbalance in Neurotransmitter Systems

Epilepsy is a common chronic neurological disorder. It is characterized by recurrent and episodic symptoms resulting from abnormal, highly synchronized neuronal discharges in the brain. Although the etiology of epilepsy is complex and diverse, the imbalance between excitation and inhibition processes in the central nervous system is widely recognized as the core pathophysiological basis for epileptogenesis [28]. This imbalance largely arises from functional disturbances in various neurotransmitter systems. Here, we review the roles and molecular mechanisms of major neurotransmitter systems in epilepsy.

#### 2.2.1 Glutamate and GABA Systems

Among various neurotransmitters, the balance between the excitatory neurotransmitter glutamate and the inhibitory neurotransmitter γ‐aminobutyric acid (GABA) is essential for maintaining normal brain function. Glutamate mediates fast excitatory synaptic transmission by activating receptors such as NMDA and AMPA. Under epileptic conditions, glutamatergic signaling becomes excessively enhanced, leading to neuronal hyperexcitability and synchronized discharges [28]. In contrast, GABA serves as the primary inhibitory neurotransmitter. It activates GABA_A receptors to promote chloride ion influx, thereby suppressing neuronal firing. Dysfunction in the GABAergic system—including reduced synthesis, impaired release, or receptor downregulation—weakens inhibitory control in neural networks and further increases excitability [28]. Studies also indicate that this excitation–inhibition imbalance extends beyond neurotransmitter levels and is closely linked to specific subtypes of GABAergic interneurons. For example, parvalbumin (PV)‐expressing interneurons play a critical role in regulating network synchronization and excitation/inhibition balance. In mouse models of Alzheimer's disease with early epilepsy, decreased PV‐positive interneurons in the hippocampus and impaired GABAergic transmission directly contribute to hippocampal hyperexcitability and elevated seizure susceptibility [29]. Moreover, GABAergic function depends on downstream signaling pathways. Phospholipase C (PLC), for instance, has been demonstrated to significantly modulate GABAergic inhibition, and its dysregulation may also be involved in epileptogenesis [30]. Therefore, a deeper understanding of the complex regulatory mechanisms of the GABAergic system is crucial for elucidating the pathological basis of epilepsy and developing novel therapeutic strategies.

#### 2.2.2 Regulatory Roles of Other Classical Neurotransmitter Systems

Beyond glutamate and GABA, other neurotransmitter systems also participate in epilepsy regulation through complex network interactions. In the cholinergic system, acetylcholine (ACh) acts as the primary stimulant of the autonomic nervous system, and its receptor dysfunctions are closely associated with epileptogenesis. Studies show that impaired function of nicotinic acetylcholine receptors (nAChRs), widely expressed in hippocampal and cortical neurons, significantly promotes epilepsy development [28]. Specifically, gene mutations encoding nAChR subunits have been identified as the cause of autosomal dominant nocturnal frontal lobe epilepsy [31]. Additionally, abnormal activation of muscarinic acetylcholine receptors (mAChRs) may enhance epileptic activity in certain brain regions. Therefore, the role of the cholinergic system in epilepsy still requires further investigation.

The monoamine neurotransmitter systems also play crucial roles in regulating epilepsy. The central histaminergic system significantly modulates neuronal excitability. Both preclinical and clinical studies have demonstrated that histamine H3 receptor antagonists, such as pitolisant, exert antiepileptic effects by enhancing the release of inhibitory neurotransmitters like GABA in the brain [32]. Meanwhile, the serotonin (5‐hydroxytryptamine, 5‐HT) system is closely linked to epilepsy and its comorbid affective disorders. In epilepsy models, alterations in the immunoreactivity of 5‐HT and its receptors, including 5‐HT1A, 5‐HT2B, and 5‐HT7, occur in brain regions such as the hippocampus. These changes suggest that serotonergic signaling disturbances contribute to epileptogenesis and related emotional problems [33]. Notably, chronic reductions in serotonin levels are considered a shared neurobiological basis for the comorbidity of epilepsy, migraine, and depression [34]. Regarding norepinephrine (NE) and dopamine (DA), the dopamine‐norepinephrine‐epinephrine cycle activates both hormonal and neuronal pathways. However, in generalized epilepsy, imbalances frequently arise among the dopaminergic, serotonergic, and noradrenergic systems. For instance, low dopamine activity combined with high serotonin activity may enhance seizure susceptibility [35]. Additionally, in chronic epilepsy models, increased immunoreactivity of the norepinephrine transporter (NET) in the cerebral cortex and medulla oblongata further confirms alterations in monoamine systems during epilepsy development [36]. Collectively, these findings highlight the significant potential of monoamine systems as therapeutic targets for epilepsy.

In summary, functional imbalance in neurotransmitter systems plays a central role in the pathogenesis of epilepsy. Disruption of the excitation/inhibition (E/I) balance, primarily involving the glutamate/GABA systems, serves as a direct driver of seizures. Meanwhile, cholinergic, monoaminergic (including histamine, serotonin, and norepinephrine), and other systems form a complex regulatory network that collectively modulates neuronal excitability. Alterations in the expression and function of receptors and ion channels, along with dysregulation of associated signaling pathways, contribute to seizure susceptibility and onset. Future research should focus on elucidating the precise interactions among these systems within specific neural circuits, as well as their connections with neuroinflammation, autophagy, and other processes. Such efforts will pave the way for developing more precise and effective therapeutic strategies for epilepsy.

### 2.3 Neuroinflammation and Immune Responses

Recent studies have increasingly demonstrated that neuroinflammation and immune responses are not merely consequences of epilepsy. Instead, they act as key pathogenic factors, driving epileptogenesis, maintaining hyperexcitable networks, and contributing to cognitive comorbidities (Figure 2).

**FIGURE 2:**
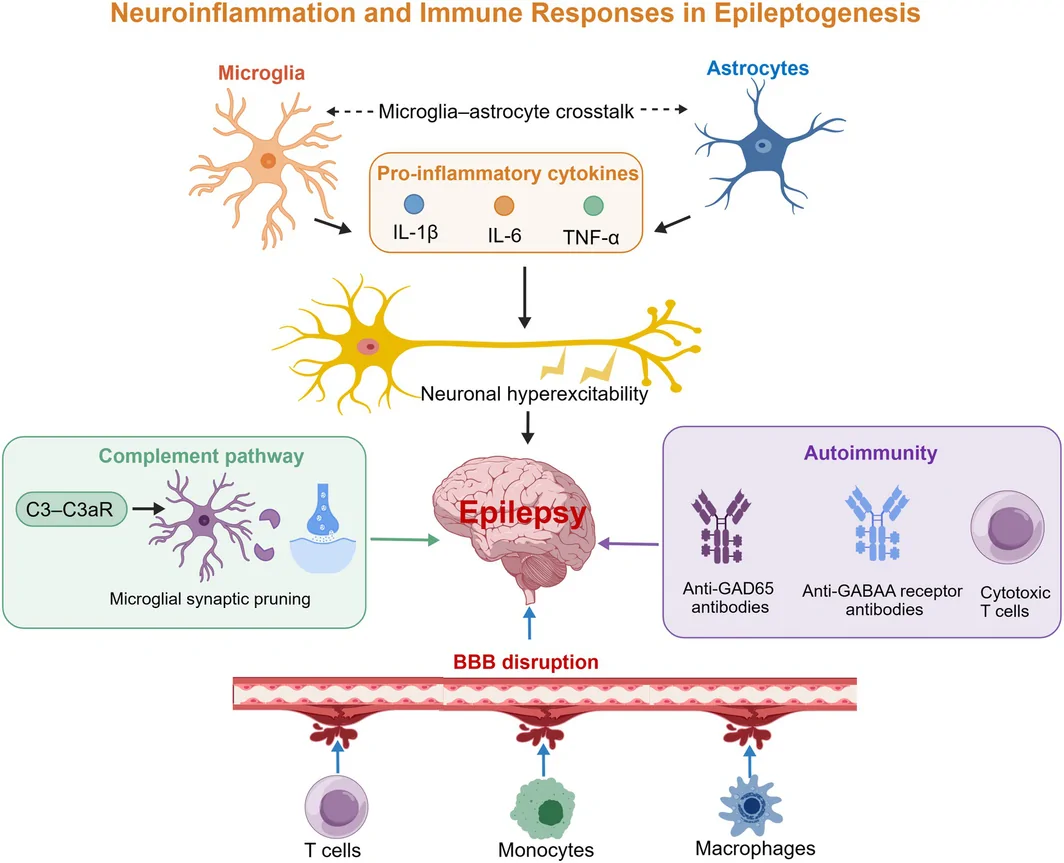
Neuroinflammation and immune‐mediated epileptogenesis. Neuroinflammation contributes to epileptogenesis through activation of microglia and astrocytes, release of pro‐inflammatory cytokines, complement activation, disruption of blood–brain barrier integrity, and infiltration of peripheral immune cells. Interactions among innate immunity, adaptive immunity, and neurovascular dysfunction promote neuronal hyperexcitability, network remodeling, and seizure susceptibility. Created with BioGDP.com [13].

#### 2.3.1 The Central Role of Neuroinflammation: Involvement of Innate and Adaptive Immunity

Neuroinflammation in epilepsy involves both intrinsic immune cells of the central nervous system (CNS) and infiltrating peripheral immune components. Microglia and astrocytes, the primary innate immune cells in the CNS, are rapidly activated following seizures. Activated microglia release pro‐inflammatory cytokines such as IL‐1β, IL‐6, and TNF‐α, as well as chemokines. These molecules directly enhance neuronal excitability and lower the seizure threshold. Furthermore, they recruit peripheral immune cells [8, 37]. Concurrently, astrocyte dysfunction—including impaired glutamate uptake and abnormal aquaporin expression—contributes to the accumulation of excitatory neurotransmitters and cerebral edema, thereby facilitating epileptogenesis [38]. In addition, aberrant contact and communication between astrocytes and microglia constitute a critical component of the neuroinflammatory circuitry [39]. Together, these mechanisms drive the sustained progression of neuroinflammation in epilepsy.

During epilepsy, disruption of the blood–brain barrier (BBB) is a common feature. This disruption allows peripheral immune cells, such as T lymphocytes and monocytes/macrophages, to enter the brain parenchyma [40, 41]. Studies have confirmed the infiltration of these cells in brain tissues from both epilepsy patients and animal models. For instance, in temporal lobe epilepsy (TLE), an increased proportion of HLA‐DR^+^ CD4^+^ T cells and alterations in monocyte subsets have been observed [42]. Moreover, adaptive immune responses are notably involved, particularly a decrease in T cell receptor (TCR) repertoire diversity. This reduction is especially prominent in patients with DRE, further indicating systemic immune dysregulation [43]. Together, these findings underscore the critical role of peripheral immune involvement in epilepsy‐related neuroinflammation.

#### 2.3.2 Key Inflammatory Mediators and Signaling Pathways

Various inflammatory molecules and pathways act as specific executors in epileptogenesis, with cytokines and chemokines playing particularly critical roles in neuroimmune mechanisms. For example, the IL‐1β/IL‐1R1 signaling pathway directly affects NMDA receptor function and disrupts neuronal chloride ion homeostasis, thereby promoting neuronal excitation. Meanwhile, TNF‐α regulates the membrane expression of AMPA receptors, which enhances excitatory synaptic transmission [44]. Additionally, IL‐17 serves as a key mediator linking innate and adaptive immunity; its levels are elevated in the body fluids and lesions of epilepsy patients. IL‐17 contributes to epileptogenesis through multiple mechanisms, including blood–brain barrier disruption and interference with neurotransmitter balance [44]. Collectively, these immune molecules form an essential foundation for neuroinflammation in driving epileptogenesis, underscoring the central importance of inflammatory regulation in epilepsy mechanisms.

Beyond cytokines, the role of the complement system in epilepsy is increasingly well‐defined, with the C3‐C3aR axis drawing particular attention. Research shows that the anti‐epileptic and cognitive‐improving effects of captopril are achieved by suppressing C3‐mediated inflammatory responses and microglial synaptic phagocytosis. Abnormal synaptic phagocytosis may further lead to pathological remodeling of neural circuits, thereby promoting epileptogenesis [39]. Moreover, in post‐traumatic epilepsy, complement system activation is considered a key driver of epilepsy development [45]. Therefore, targeting complement signaling not only reveals novel pathological mechanisms but also provides potential strategies for epilepsy prevention and treatment.

Regarding specific immune pathways, the CD1d/NKT cell axis has been identified as a key driver of cortical hyperexcitability and epileptogenesis in post‐traumatic epilepsy models. Mice deficient in CD1d or NKT cells show markedly reduced epilepsy susceptibility and spontaneous seizure frequency. Statins such as simvastatin demonstrate potential for epilepsy prevention by disrupting this axis during the early post‐traumatic phase [46]. Meanwhile, the Toll‐like receptor pathway significantly contributes to the link between neuroinflammation and epilepsy. Glial cells detect damage‐associated molecular patterns via pattern recognition receptors including TLR and NLR. This activates downstream signaling pathways such as NF‐κB and promotes pro‐inflammatory cytokine production [37]. In tuberous sclerosis complex‐related epilepsy, exosome‐carried molecules like miR‐142‐3p can also induce neuroinflammatory cascades through TLR7/8 activation [47]. Together, these mechanisms reinforce the strong connection between neuroinflammation and seizure generation. They indicate that targeting specific immune pathways may offer effective strategies for epilepsy prevention and treatment.

#### 2.3.3 Autoimmunity and Specific Epilepsy Syndromes

A subset of epilepsies is clearly caused by autoimmune diseases, including anti‐GAD65 antibody‐associated epilepsy and anti‐GABA A receptor encephalitis. In these disease processes, autoantibodies or cytotoxic T cells directly and persistently attack neuronal surface or intracellular antigens. This leads to neuronal dysfunction, abnormal increases in excitability, and even cell death [48, 49]. Therefore, a deep understanding of these immune mechanisms is crucial for the precise diagnosis and treatment of epilepsy. Notably, T cell‐mediated neuronal killing targeting GAD65 is considered the core pathological mechanism of this type of temporal lobe epilepsy, rather than complement‐mediated cell destruction [49]. Meanwhile, new‐onset refractory status epilepticus and its subtype in children—febrile infection‐related epilepsy syndrome—are also closely associated with abnormal activation of the innate immune system [50, 51]. Early recognition and intervention for these autoimmune‐related epilepsies are particularly critical.

Neuroinflammation and immune responses are central to the onset and progression of epilepsy. The activation of glial cells in the brain and the infiltration of peripheral immune cells contribute to this process. Additionally, classic cytokine storms, complement‐mediated synaptic pruning, and specific autoimmune attacks form a complex immune network that underlies the pathology of epilepsy. Future research will continue to elucidate the interactions between these pathways and promote the development of precise treatment strategies targeting neuroimmune mechanisms. These advances offer hope for improving the prognosis of epilepsy patients, especially those with drug‐resistant epilepsy.

### 2.4 Cellular Homeostasis Imbalance in Epilepsy

The onset and progression of epilepsy, known as epileptogenesis, constitute a complex process involving alterations in multiple cellular and molecular mechanisms. Among these, the disruption of cellular homeostasis—which maintains the internal stability of cells—plays a critical role in the pathophysiology of epilepsy. This imbalance particularly involves dysregulation in essential cellular processes such as autophagy, apoptosis, and ferroptosis.

#### 2.4.1 Autophagy: The Dual Role in Epilepsy

Autophagy is an evolutionarily conserved degradation process. It delivers cytoplasmic components, such as damaged organelles and misfolded proteins, to lysosomes for degradation via the formation of autophagosomes. Thus, it is essential for maintaining neuronal structural and functional homeostasis [52]. In epilepsy, autophagy exhibits a dual role. On one hand, it may exert protective effects by clearing abnormal protein aggregates and damaged mitochondria induced by seizure stress. This helps maintain intracellular homeostasis and suppress neuronal hyperexcitability. Studies have shown that in the brains of epileptic model rats, autophagy‐related markers such as the LC3‐II/LC3‐I ratio and Beclin‐1 protein levels increase significantly during the early phase (12–48 h) after seizures. Concurrently, the level of the autophagy substrate P62 decreases, indicating activation of the autophagic flux [53]. This early activation may represent a compensatory mechanism against epilepsy‐induced cellular stress. Therefore, moderate autophagy likely confers neuroprotective potential in the early stages of epilepsy.

On the other hand, excessive or dysfunctional autophagy may promote neuronal death and exacerbate epileptogenesis. One of the major regulatory pathways controlling autophagy is the mTOR pathway, and its abnormal activation is linked to various epileptic syndromes [54]. Moreover, dysregulation of autophagy involves the role of microRNAs (miRNAs). Specific molecules termed “autophagy miRNAs” can modulate the expression of autophagy‐related genes at the post‐transcriptional level, thereby establishing a novel mechanism for epileptogenesis [55]. For example, in Lafora disease, a progressive myoclonus epilepsy, mutations in the laforin or malin genes lead to impaired autophagy, decreased proteasome activity, and endoplasmic reticulum stress. This disrupts protein homeostasis and is accompanied by marked oxidative stress, ultimately collectively aggravating disease progression [56, 57]. These findings emphasize the complexity and importance of autophagy regulation in epilepsy and related disorders.

#### 2.4.2 Apoptosis: The Executor of Neuronal Loss in Epilepsy

Apoptosis serves as the primary form of programmed cell death and plays a central role in epilepsy‐induced neuronal loss. During epileptic seizures, glutamate receptor‐mediated excitotoxicity triggers intracellular calcium overload and mitochondrial dysfunction. This activation then promotes pro‐apoptotic proteins, including p53, and initiates the caspase cascade, ultimately leading to neuronal apoptosis [54]. Therefore, a deeper understanding of apoptosis regulation is essential for developing novel therapeutic strategies for epilepsy. Notably, autophagy and apoptosis exhibit close spatiotemporal interactions during epilepsy progression. Li et al. demonstrated that in developmental brain epilepsy models, autophagy activation, reflected by an increased LC3‐II/I ratio, occurs earlier than significant apoptosis. The latter is characterized by reduced neuronal numbers and increased apoptotic cells, accompanied by a subsequent decline in anti‐apoptotic Bcl‐2 protein levels [53]. This finding implies that early autophagy may attempt to rescue damaged cells, but when injury becomes severe, cell fate shifts toward apoptosis. Such a temporal relationship suggests that autophagy could provide an early window for intervening in neuronal damage. Moreover, sustained epileptic stress and endoplasmic reticulum stress are also recognized as key pathways triggering neuronal apoptosis, particularly in genetic epilepsies [58]. These stress responses exacerbate neuronal injury and loss through multiple molecular mechanisms. Targeting these pathways may offer new approaches to reduce epilepsy‐related neuronal loss.

#### 2.4.3 Ferroptosis: An Emerging Focus in Epilepsy Research

Ferroptosis is an iron‐dependent form of regulated cell death, characterized by abnormal accumulation of lipid peroxides. Recent studies have confirmed its close association with the onset and progression of epilepsy. In patients with drug‐resistant epilepsy, such as temporal lobe epilepsy (TLE), significant iron and lipid peroxide accumulation has been observed in brain tissues [59, 60]. Single‐cell transcriptomic analysis further revealed dysregulated iron metabolism in microglia from TLE patients. This dysregulation manifests as increased iron accumulation, upregulation of ferritin synthesis, and enhanced metabolic activity related to oxidative damage, collectively forming a distinct phenotype closely linked to neuroinflammation. The abundance of these so‐called “ferroptosis‐associated microglia” positively correlates with seizure frequency [59], suggesting their potential role in epilepsy progression. Evidence of ferroptosis involvement has also been observed in animal models. For instance, in a soman‐induced epilepsy model, iron‐laden glial cells and ferritin levels were elevated, while treatment with the selective inducible nitric oxide synthase (iNOS) inhibitor 1400 W alleviated iron dyshomeostasis and ameliorated associated brain pathology and functional impairment [61]. Moreover, in epileptic mouse brain tissues, both peroxynitrite (ONOO^−^), a potent oxidant, and tissue viscosity were elevated, each closely associated with ferroptosis progression [62]. Together, these findings support the important role of ferroptosis in the pathological mechanisms of epilepsy and provide a theoretical basis for exploring new therapeutic targets.

#### 2.4.4 Interactions and Therapeutic Prospects

Autophagy, apoptosis, and ferroptosis do not function in isolation; instead, they are interwoven into a complex network regulating cell fate. Autophagy may suppress apoptosis and ferroptosis by clearing damaged components. However, its dysregulation can also trigger or worsen these processes. Mitochondria serve as a common hub, and their dysfunction leads to reactive oxygen species (ROS) bursts and calcium homeostasis imbalance. These events link the three processes and drive neuronal excitotoxicity, neuroinflammation, and ultimately cell death [63]. Neuroinflammation, particularly the response driven by reactive astrocytes and microglia, is not only a consequence of epilepsy but also actively disrupts cellular homeostasis through pro‐inflammatory cytokine release. This, in turn, exacerbates autophagy, apoptosis, and ferroptosis [57, 64]. Thus, the interactions among these processes hold a central role in epilepsy pathology.

In summary, cellular homeostasis is fundamental to normal brain function. During the onset and progression of epilepsy, autophagy, apoptosis, and ferroptosis become dysregulated under stress. These processes interact and form a complex network, collectively determining neuronal fate—whether neurons survive, become dysfunctional, or undergo cell death. Elucidating the molecular details of these mechanisms and their interconnections will establish a solid theoretical foundation for developing novel biomarkers and neuroprotective therapies targeting specific cell death pathways. Based on this, future clinical treatments—especially for patients with drug‐resistant epilepsy—are expected to gain new intervention strategies and renewed hope.

### 2.5 Epigenetic Regulation

In recent years, a growing number of studies have revealed that epigenetic regulation plays a critical role in epileptogenesis and the maintenance of chronic epilepsy [3]. Epigenetic mechanisms do not alter the DNA sequence; instead, they regulate gene expression and influence the balance between excitation and inhibition in neural networks, thereby participating in the pathological processes of epilepsy. Key epigenetic mechanisms include histone modifications, DNA methylation, non‐coding RNAs, N6‐methyladenosine (m6A) methylation, and regulation by the transcription factor REST/NRSF.

#### 2.5.1 Histone Modifications

Histone modifications represent a core aspect of epigenetic regulation. They primarily alter chromatin structure through mechanisms such as acetylation and methylation, thereby influencing gene transcription. However, in the epileptic state, this dynamic balance is often disrupted. Studies have revealed widespread abnormalities in histone acetylation levels within brain tissues of epilepsy patients and animal models, particularly in the hippocampal region [65]. These findings underscore the critical role of histone acetylation in epileptogenesis. Specifically, the expression and activity of histone deacetylases (HDACs) exhibit significant fluctuations during epilepsy development [66]. For instance, after status epilepticus, the expression of HDAC subtypes such as HDAC1, 2, 3, and 11 initially decreases rapidly, followed by a general upregulation during the latent phase of the disease [67]. These dynamic changes are closely associated with altered expression of epilepsy‐related genes, including neurotrophic factors, ion channels, and inflammatory factors. Notably, HDAC inhibitors, such as valproate, have demonstrated anti‐seizure and even anti‐epileptogenic potential in preclinical models [3]. This provides compelling evidence for therapeutic strategies targeting histone modifications in epilepsy.

#### 2.5.2 DNA Methylation

DNA methylation involves adding a methyl group to cytosine in DNA molecules, and this modification typically leads to gene silencing. In patients with temporal lobe epilepsy and hippocampal sclerosis (TLE‐HS), the whole‐genome DNA methylation profile shows significant alterations [68, 69]. These changes are etiology‐ and brain region‐specific [70]. Therefore, DNA methylation may play a crucial role in TLE pathogenesis. For instance, hippocampal tissues from TLE‐HS patients contain numerous differentially methylated regions. These regions involve genes related to neurodevelopment, synaptic function, and inflammatory responses [68]. Additionally, hypermethylation of the Reelin gene promoter is associated with granule cell dispersion, a typical pathological feature of TLE [71]. Moreover, the expression of DNA methyltransferases, such as DNMT1 and DNMT3A, is altered in epileptic brain tissues. This alteration further drives the formation of abnormal DNA methylation patterns [72]. These epigenetic regulatory mechanisms collectively contribute to the neuropathological process of epilepsy. Notably, the DNA methylation profile in peripheral blood can reflect epigenetic changes in the brain. This suggests its potential as a biomarker for epilepsy [73, 74]. Thus, it offers a new direction for non‐invasive diagnosis and monitoring of the disease.

#### 2.5.3 Non‐Coding RNAs

Dysregulation of non‐coding RNAs (miRNAs, lncRNAs) in epilepsy is closely associated with disease progression. Various miRNAs, such as miR‐134, miR‐146a, and miR‐132, exhibit abnormal expression in epilepsy models, as well as in the brain tissues and blood of patients [75, 76]. Consequently, these miRNAs profoundly influence epilepsy progression by targeting gene networks involved in synaptic plasticity, neuroinflammation, neuronal death, and ion channels [77]. For instance, inhibition of miR‐134 has been demonstrated to exert potent anticonvulsant and antiepileptogenic effects [77]. Collectively, these findings underscore the significant role of miRNAs in epilepsy mechanisms. Meanwhile, lncRNAs such as GAS5 and circular RNAs (circRNAs) also participate in the regulatory network of epilepsy. They operate through complex competing endogenous RNA (ceRNA) mechanisms, thereby influencing neuronal excitability and epileptogenesis [78, 79]. This mechanism further expands our understanding of the functional diversity of non‐coding RNAs in neurological diseases.

#### 2.5.4 m6A

In recent years, growing evidence has highlighted the critical role of m6A in the pathogenesis and progression of epilepsy. For instance, transcriptome‐wide m6A modifications are significantly dysregulated in hippocampal tissues from humans and mice with temporal lobe epilepsy [80]. These alterations in m6A levels are closely linked to key cellular pathways, such as metabolism and autophagy. Thus, aberrant m6A modifications may constitute a fundamental molecular basis for epileptogenesis. In animal models, elevating m6A levels effectively reduces susceptibility to epilepsy and mitigates its severity [80]. Further studies demonstrate that the fat mass and obesity‐associated gene (FTO), an m6A demethylase, alleviates post‐traumatic epilepsy by upregulating NR4A2 expression. This effect occurs through an m6A‐YTHDF2‐dependent mechanism [81]. Collectively, these findings indicate that m6A not only unveils a novel layer of gene expression regulation but also provides a crucial entry point for investigating epilepsy pathology. Future in‐depth research on the m6A regulatory network holds promise for developing new therapeutic strategies against epilepsy.

#### 2.5.5 REST/NRSF

REST/NRSF is a key transcriptional repressor. It plays a critical role in nervous system development and gene expression regulation. This factor maintains normal nervous system function by regulating downstream target genes. Thus, stable expression and activity of REST are essential for neuronal homeostasis. In hyperexcitable pathological states, such as epilepsy, REST becomes abnormally activated. It then recruits co‐repressor complexes, including histone deacetylases (HDACs). Consequently, REST represses a series of target genes containing RE1 elements [82, 83]. These target genes include key genes encoding ion channels, neurotransmitter receptors, and synaptic proteins. Their downregulation disrupts the balance between neuronal excitation and inhibition. Ultimately, this promotes epileptogenesis and network remodeling [3]. Therefore, abnormal REST activation profoundly impacts neuronal function. Given these mechanisms, REST is regarded as a core molecule linking neuronal activity to epigenetic regulation. It also emerges as a potential target for therapeutic intervention. Further elucidating its role in epilepsy will aid in developing novel treatment strategies.

Epigenetic regulation is increasingly recognized as a key interface linking genetic susceptibility to downstream pathogenic processes in epilepsy. Histone modifications, DNA methylation, non‐coding RNAs, m6A methylation, and REST/NRSF collectively influence ion channel expression, neurotransmitter signaling, neuroinflammatory responses, metabolic homeostasis, and neuronal survival. Through these interconnected regulatory networks, epigenetic mechanisms shape multiple stages of epileptogenesis, from early molecular alterations to network remodeling and chronic disease progression. Importantly, their ability to simultaneously modulate diverse pathogenic pathways positions epigenetic regulation as a promising source of biomarkers and therapeutic targets. Emerging approaches, including HDAC inhibition, RNA‐based therapies, and modulation of m6A‐dependent signaling, may provide new opportunities for precision interventions, particularly in patients with drug‐resistant epilepsy.

### 2.6 Metabolic Abnormalities and Mitochondrial Dysfunction

Accumulating evidence indicates that metabolic abnormalities and mitochondrial dysfunction contribute not only to epileptogenesis but also to treatment resistance. As key regulators of neuronal energy homeostasis and cellular stress responses, they have emerged as important therapeutic targets in epilepsy [84].

#### 2.6.1 Mitochondrial Dysfunction Is the Pathophysiological Core of Epilepsy

Mitochondria are essential for maintaining neuronal energy homeostasis and excitability. Energy metabolism crisis and neuronal excitability imbalance represent key mechanisms in epileptogenesis, as high‐frequency neuronal discharges are highly energy‐consuming processes that require continuous energy supply. If mitochondrial electron transport chain function is impaired, it causes insufficient ATP production; this subsequently fails to sustain normal ion channel functions, such as the sodium‐potassium pump. Consequently, cell membrane depolarization occurs, the neuronal firing threshold is lowered, and network excitability ultimately increases [84]. Clinical studies further confirm that in drug‐resistant epilepsy patients, local cerebral glucose hypometabolism detected by positron emission tomography directly correlates with significantly reduced mitochondrial complex IV activity, thereby demonstrating that cellular energy failure underlies the potential mechanism of drug‐resistant epilepsy [85]. Thus, improving energy metabolism may help control abnormal elevations in neuronal excitability.

Oxidative stress injury also plays a critical role in the progression of epilepsy, as mitochondria serve as the primary source of reactive oxygen species (ROS). When the electron transport chain function is impaired, it leads to excessive ROS production. Furthermore, excessive ROS attacks lipids, proteins, and DNA, causing neuronal damage and death. It also disrupts the function of ion channels and neurotransmitter receptors, thereby directly or indirectly enhancing neuronal excitability and promoting epileptogenesis [63]. Additionally, this oxidative stress forms a vicious cycle with calcium homeostasis disruption, collectively exacerbating neuronal damage and hyperexcitability. Thus, antioxidant strategies may offer a new direction for epilepsy treatment.

Dysregulation of calcium homeostasis is closely linked to the pathological mechanisms of epilepsy, as mitochondria act as key intracellular calcium buffers, and their impaired function markedly reduces calcium buffering capacity. During epileptic seizures, glutamate‐mediated excitotoxicity causes a sharp rise in intracellular calcium levels, which then induces mitochondrial calcium overload. Excessively high calcium concentrations not only activate apoptotic pathways but also disrupt mitochondrial membrane potential, and they exacerbate energy crisis and ROS production, ultimately leading to neuronal death [63]. This process underscores the central role of calcium homeostasis in neuronal damage in epilepsy.

Imbalance in mitochondrial dynamics significantly influences epilepsy pathogenesis, as the dynamic equilibrium between mitochondrial fusion and fission is essential for maintaining their function and health. Studies reveal that in temporal lobe epilepsy, downregulated expression of the mitochondrial transport protein TRAK1 elevates the mitochondrial fission factor MFF, thereby inducing excessive mitochondrial fission. This disruption between fission and fusion not only compromises mitochondrial function but also heightens epilepsy susceptibility [86]. Furthermore, mutations in dynamin‐related protein 1 are demonstrated to cause mitochondrial fission defects and trigger DRE, which further emphasizes the critical role of mitochondrial dynamics in epileptogenesis [87]. Consequently, preserving mitochondrial dynamic balance emerges as a potential therapeutic target.

#### 2.6.2 Close Link Between Metabolic Abnormalities and Epilepsy

Metabolic disturbances are increasingly recognized as important contributors to epilepsy. Impaired glucose utilization, mitochondrial energy deficits, and abnormalities in amino acid and intermediary metabolism can disrupt neuronal bioenergetics, alter neurotransmitter homeostasis, and increase seizure susceptibility. Classical examples include glucose transporter type 1 deficiency syndrome and pyruvate dehydrogenase complex deficiency, both of which impair cerebral energy supply and are frequently associated with DRE [88]. More broadly, inherited metabolic disorders often converge on common downstream mechanisms, including mitochondrial dysfunction, oxidative stress, neuroinflammation, and excitation–inhibition imbalance, highlighting the central role of metabolic homeostasis in epileptogenesis [89, 90, 91].

Metabolic and mitochondrial dysfunction extends beyond energy failure and represents a critical point of convergence among multiple epileptogenic pathways. Impaired ATP production, excessive ROS generation, and calcium dysregulation not only enhance neuronal excitability but also promote neuroinflammatory signaling, disrupt cellular homeostasis, and increase susceptibility to ferroptotic and apoptotic cell death. Conversely, inflammatory mediators and persistent seizure activity further exacerbate mitochondrial dysfunction, creating a self‐reinforcing cycle of metabolic stress and neuronal injury. These observations position mitochondrial dysfunction as a central hub linking neurotransmitter imbalance, neuroinflammation, regulated cell death, and network remodeling. Consequently, therapeutic strategies targeting mitochondrial homeostasis and metabolic resilience may provide broader disease‐modifying effects than approaches directed at individual downstream pathways.

### 2.7 Neural Networks and the Neurovascular Unit

In recent years, research perspectives have expanded beyond purely neuronal abnormalities. They now encompass multidimensional mechanisms involving neural network remodeling, neurovascular unit dysfunction, and blood–brain barrier disruption. These factors are interconnected and collectively contribute to the onset, progression, and treatment resistance of epilepsy.

#### 2.7.1 Neural Network Remodeling and Connectome Abnormalities

Epilepsy is fundamentally a “network disorder,” characterized by functional and structural remodeling of large‐scale neural networks. Studies on deep brain stimulation (DBS) for drug‐resistant epilepsy reveal that its mechanism involves a common cortico‐subcortical network. Key nodes of this network include the anterior cingulate cortex, posterior cingulate cortex, medial prefrontal cortex, sensorimotor cortex, insula, and subcortical structures such as the basal ganglia, midbrain, basal forebrain, and cerebellum. Notably, the anterior thalamic nucleus is identified as a central hub of this network, exhibiting the highest betweenness and closeness centrality. The caudate nucleus and mammillothalamic tract also demonstrate high centrality values [92]. This network overlaps with known epileptic circuits and brain regions involved in seizure propagation and generalization. This suggests that the antiepileptic effects of DBS may partly arise from modulation of this shared network. Furthermore, neural network remodeling occurs not only at the macroscopic connection level but also involves activity‐dependent changes in microstructure. For example, in generalized absence epilepsy models, seizures themselves drive increased oligodendrogenesis and myelination—termed “maladaptive myelination.” This abnormal myelination, in turn, enhances synchrony within thalamocortical networks, thereby promoting epilepsy progression. Further studies indicate that blocking such activity‐dependent myelination reduces seizure burden and decreases ictal synchronization [93]. Additionally, during the interictal period, neural network dysfunction is considered a key basis for cognitive comorbidities in epilepsy patients. Pathological network interactions span different spatial and temporal scales [94]. Therefore, a deeper understanding of the dynamic changes in neural networks across multiple scales is essential for comprehensively elucidating the pathological mechanisms of epilepsy.

#### 2.7.2 Dysfunction of the Neurovascular Unit and Disruption of the Blood–Brain Barrier

The neurovascular unit is a critical structure for maintaining brain homeostasis, and its components include brain microvascular endothelial cells, astrocytic end‐feet, pericytes, neurons, and the extracellular matrix. The blood–brain barrier represents the core functional element of this unit, and its impaired integrity plays a vital role in the initiation and progression of epilepsy. Substantial evidence indicates that after insults such as traumatic brain injury, structural damage to the blood–brain barrier leads to extravasation of blood‐derived components like albumin into the brain parenchyma. This albumin is recognized by TGFβR2 receptors on astrocytes, triggering astrocyte transformation and functional abnormalities, which ultimately result in neuronal hyperexcitability and epileptogenesis [95]. This mechanism is not only observed in post‐traumatic epilepsy but may also represent a common pathway in other acquired forms of epilepsy [96]. Notably, blood–brain barrier disruption is not merely a consequence of epilepsy; it acts as an active pathogenic factor. Seizures can further increase barrier permeability, thereby establishing a vicious cycle of “blood‐brain barrier disruption–seizures–further barrier disruption” [97]. At the cellular level, glutamate released during seizures activates cytosolic phospholipase A2 in cerebrovascular endothelial cells. This activation promotes overexpression of drug efflux transporters, such as P‐glycoprotein and breast cancer resistance protein, at the blood–brain barrier. This process is a key mechanism underlying drug‐resistant epilepsy [98, 99] and further aggravates disturbances in the brain's internal environment. Moreover, hemichannels composed of connexin 43 in astrocytes are activated in epilepsy, and their inhibitor TAT‐Gap19 has been demonstrated to reduce blood–brain barrier permeability and decrease seizure burden, underscoring its crucial role in regulating barrier function and modulating seizure activity [100]. Therefore, targeting the regulation of blood–brain barrier function holds promise for developing novel interventional strategies in epilepsy treatment.

#### 2.7.3 Neuro‐Immune‐Vascular Interactions and Epilepsy Progression

The interplay among neural networks, the immune system, and the vascular system collectively shapes the complex pathophysiology of epilepsy. Disruption of the blood–brain barrier opens pathways for peripheral immune cells, such as monocytes and T lymphocytes, to infiltrate the central nervous system [41]. These infiltrating cells interact with resident microglia and astrocytes, promoting the release of pro‐inflammatory cytokines such as IL‐1β, IL‐6, and TNF‐α and thereby amplifying neuroinflammatory responses. As key components of the neurovascular unit, pericytes are highly sensitive to inflammatory signaling, and their dysfunction further compromises blood–brain barrier integrity, perpetuating neurovascular pathology and epilepsy progression [101]. This dysregulation is particularly evident in aging‐associated epilepsy, where cerebrovascular dysfunction, blood–brain barrier decline, and chronic low‐grade inflammation collectively increase seizure susceptibility and may interact with neurodegenerative processes [102]. Importantly, blood–brain barrier dysfunction, immune activation, and neural network remodeling form a mutually reinforcing neural–immune–vascular axis, illustrating how neurovascular and inflammatory processes become integrated during epileptogenesis.

### 2.8 Mechanistic Crosstalk and Convergence in Epileptogenesis

Although the pathogenic mechanisms discussed above are presented separately for clarity, epileptogenesis is driven by extensive crosstalk among these pathways rather than by isolated molecular events. Genetic and ion channel abnormalities initiate neuronal hyperexcitability, whereas neurotransmitter imbalance, neuroinflammation, metabolic dysfunction, cellular homeostasis disruption, and epigenetic dysregulation collectively amplify and sustain this process. Among these mechanisms, neuroinflammation appears to function as a central amplifier, linking excitation–inhibition imbalance, mitochondrial dysfunction, oxidative stress, and regulated cell death. Conversely, ROS accumulation and ferroptotic or apoptotic injury promote the release of DAMPs, which further activate glial cells, amplify inflammatory responses, and contribute to blood–brain barrier dysfunction.

At a broader systems level, these molecular and cellular abnormalities converge on a neural–immune–vascular axis characterized by neural network remodeling, blood–brain barrier dysfunction, and persistent neuroinflammation (Figure 2). These interconnected processes establish self‐reinforcing pathogenic circuits that sustain hyperexcitability, promote epileptogenesis, and contribute to therapeutic resistance. This integrated framework helps explain why many single‐target interventions show limited efficacy and supports the development of multi‐target, network‐oriented, and precision therapeutic approaches for epilepsy.

## 3 Challenges in Epilepsy Treatment

Although the molecular mechanisms of epilepsy are discussed separately in the preceding sections, they function as a highly interconnected pathogenic network in vivo. Genetic susceptibility, neurotransmitter imbalance, neuroinflammation, epigenetic remodeling, metabolic dysfunction, disturbances in cellular homeostasis, and neurovascular abnormalities collectively contribute to neuronal hyperexcitability, network remodeling, and epileptogenesis. This mechanistic convergence helps explain the marked heterogeneity of epilepsy and the limited efficacy of many single‐target interventions, highlighting the need for precision and multi‐target therapeutic strategies.

Despite growing insights into the molecular and cellular mechanisms underlying epileptogenesis, translating these discoveries into effective clinical interventions remains challenging. Current epilepsy management continues to face several major obstacles, including drug‐resistant epilepsy (DRE), adverse drug effects, comorbidity management, limitations in precision diagnosis and treatment, and SUDEP (Figure 3, left).

**FIGURE 3:**
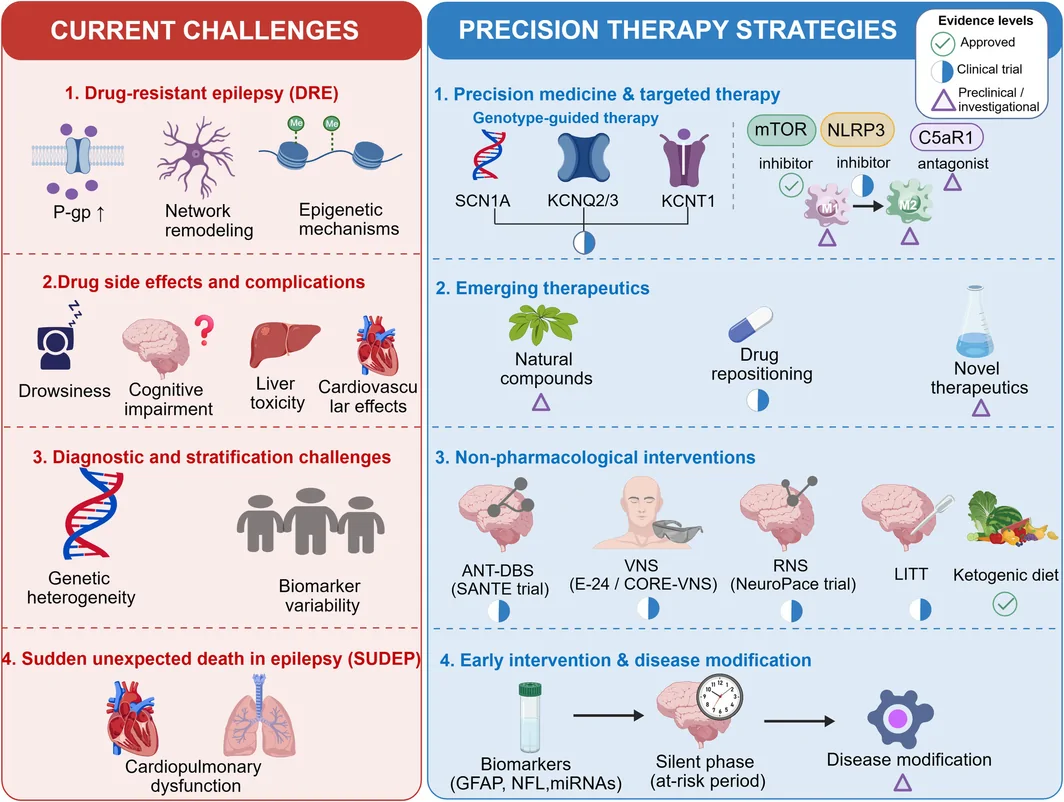
Current challenges and precision therapeutic strategies in epilepsy. Major challenges in epilepsy management include drug‐resistant epilepsy (DRE), treatment‐related adverse effects, patient heterogeneity, and sudden unexpected death in epilepsy (SUDEP). Emerging precision therapeutic approaches include genotype‐guided therapy, pathway‐targeted interventions, drug repurposing, neuromodulation, dietary therapies, and surgical strategies. Integration of biomarkers, patient stratification, and early intervention may facilitate disease modification and support the transition toward precision epilepsy care. Created with BioGDP.com [13].

### 3.1 Drug‐Resistant Epilepsy

Despite the development of numerous new ASMs over recent decades, approximately 33% of epilepsy patients do not respond to existing drug therapies and develop DRE. This represents the most formidable challenge in epilepsy treatment [2]. DRE not only severely impairs patients' quality of life but also significantly increases risks of premature death, accidental injuries, and neuropsychiatric comorbidities. Furthermore, it imposes substantial economic and caregiving burdens on families and society [103]. The mechanisms of DRE are complex and multifaceted, involving molecular, cellular, network, and systemic levels. However, these mechanisms remain incompletely understood, and several core hypotheses have been proposed.

#### 3.1.1 Target Hypothesis

According to this hypothesis, the molecular targets of antiseizure medications (ASMs)—such as ion channels and neurotransmitter receptors—may undergo structural and functional alterations during chronic epilepsy development and recurrent seizures. These changes can prevent the drugs from working effectively. For instance, voltage‐gated sodium channels are common targets for multiple first‐line ASMs, including carbamazepine and lamotrigine. Studies indicate that in chronic epilepsy models, depletion of intracellular polyamines (e.g., spermine) weakens use‐dependent sodium channel blockade, thereby reducing the efficacy of related drugs [10]. Thus, adaptive changes in target function may contribute to diminished drug response. Genetic studies provide further support: loss‐of‐function mutations in the SCN1A gene, a major cause of developmental and epileptic encephalopathies such as Dravet syndrome, lead to Nav1.1 sodium channel dysfunction. This has been identified as a key mechanism of drug resistance at the target level [104]. Moreover, mutations in other targets, such as GABAA receptor subunits, can impair the efficacy of benzodiazepines and other agents, resulting in innate or acquired drug resistance. These findings suggest that target alterations may interact with broader genetic and network‐level mechanisms to influence treatment response in epilepsy.

#### 3.1.2 Transporter Hypothesis

This hypothesis represents one of the extensively investigated drug resistance mechanisms. It proposes that in the lesioned brain tissues of patients with DRE—such as hippocampal sclerosis and cortical dysplasia—various drug efflux transporters on the blood–brain barrier and neuronal membranes are overexpressed. Notably, these include ABC transporter superfamily members like P‐glycoprotein (P‐gp) and multidrug resistance‐associated proteins (MRPs). These transporters act as “molecular pumps,” actively extruding ASMs from cells. Consequently, they reduce effective drug concentrations in lesioned areas and contribute to drug resistance [11]. Thus, this mechanism holds significant pathological importance in impairing local drug distribution. Genetic variation and epigenetic regulation of ABC transporters may influence their expression and function within the central nervous system, thereby affecting local ASM distribution and treatment response [105]. Furthermore, animal model studies demonstrate that P‐gp inhibitors can reverse resistance to certain ASMs, providing experimental support for this hypothesis and suggesting that transporter‐targeted interventions may have therapeutic potential.

#### 3.1.3 Network Hypothesis

Epilepsy is not merely a disorder of abnormal neuronal discharges; it is increasingly understood as a “brain network disorder.” This hypothesis posits that prolonged and recurrent seizures induce large‐scale plastic reorganization of neural networks. Consequently, an aberrant, highly synchronized, and self‐sustaining “epileptogenic network” emerges. Although medications can suppress localized abnormal neuronal firing, they often fail to control the widespread and dynamic activity of such pathological networks. Moreover, the development of drug tolerance may not only relate to reduced drug efficacy but also involve alterations in interregional brain connectivity and shifts in the excitatory‐inhibitory balance within specific brain areas [12]. Therefore, understanding epilepsy's network mechanisms is crucial for developing more effective therapeutic strategies. Functional neuroimaging studies—such as those using resting‐state functional MRI or single‐photon emission computed tomography—have confirmed characteristic alterations in cortico‐subcortical perfusion networks or functional connectivity in patients with DRE. These network features may even serve as biomarkers for predicting treatment response [106]. Additionally, studies on canine models of DRE support this network hypothesis, indicating that network‐level alterations are key factors influencing the efficacy of antiseizure medications [107]. Collectively, multifaceted evidence underscores the central role of epileptic networks in disease progression.

#### 3.1.4 Epigenetic Mechanisms and Emerging Hypotheses

In recent years, the role of epigenetic regulation in epileptogenesis and drug resistance mechanisms has garnered increasing attention. Epigenetic mechanisms modulate gene expression without altering the DNA sequence, thereby offering novel insights into the long‐term plastic changes observed in DRE. Multiple epigenetic mechanisms—including histone modifications (e.g., acetylation and methylation), DNA methylation, non‐coding RNAs (such as miR‐4465), and m6A RNA methylation—have been shown to precisely regulate the expression of numerous epilepsy‐associated genes. These modifications subsequently influence key pathophysiological processes, such as neuronal excitability, synaptic plasticity, glial cell function, and neuroinflammation, ultimately contributing to the development of drug resistance [3, 108]. For instance, aberrant expression of histone deacetylases (HDACs) may promote drug tolerance by disrupting genes involved in neural plasticity. These findings suggest that epigenetic regulation may provide a mechanistic link between neuronal hyperexcitability, neuroinflammation, and long‐term treatment resistance.

Furthermore, the emerging intrinsic severity hypothesis reconceptualizes DRE as a failure of endogenous homeostatic mechanisms to maintain network stability in the presence of persistent pathogenic stressors [109, 110]. This perspective emphasizes that drug resistance may reflect the overall severity and complexity of the epileptogenic network rather than alterations in a single molecular target. Consistent with this view, therapeutic strategies targeting convergent pathogenic mechanisms, particularly neuroinflammation and blood–brain barrier dysfunction, have attracted increasing attention. For example, modulation of the purinergic P2X7 receptor has emerged as a promising approach for restoring neuroimmune homeostasis and improving treatment responsiveness in DRE [111]. More broadly, DRE is increasingly recognized as a multifactorial disorder arising from interacting mechanisms rather than a single pathogenic process. The target, transporter, network, and epigenetic hypotheses provide complementary explanations for drug resistance at molecular, pharmacokinetic, systems, and regulatory levels, converging through shared processes such as neuroinflammation, blood–brain barrier dysfunction, and maladaptive network remodeling. Precision therapeutic approaches, including neuromodulation strategies and multi‐omics‐guided personalized medicine, have therefore attracted increasing interest as potential solutions to the limitations of conventional ASM‐based treatment [111, 112]. This integrated perspective may help explain the limited efficacy of many single‐target interventions and supports the development of disease‐modifying therapeutic strategies that simultaneously address multiple convergent mechanisms.

### 3.2 Drug Side Effects and Complications

Currently available ASMs often provide only symptomatic control. They may cause various adverse effects, including drowsiness, dizziness, cognitive impairment, and abnormal liver function. These side effects significantly impact patients' quality of life and treatment adherence [2]. Moreover, epilepsy is frequently accompanied by multiple comorbidities. The management of these conditions also poses significant challenges in clinical therapy. Therefore, there is an urgent need to develop safer and more targeted treatment strategies.

Among the numerous comorbidities of epilepsy, cognitive and behavioral deficits are particularly common. Traditionally, seizures and epileptiform discharges have been regarded as the primary causes of cognitive impairment. However, recent studies indicate that the underlying etiology of epilepsy and the resulting abnormalities in neuronal dynamics—specifically, the disruption of neural information processing timing—represent important mechanisms for cognitive deficits, independent of seizures [113]. Meanwhile, psychiatric comorbidities are highly prevalent in epilepsy patients. The incidence of depression, anxiety, and psychotic symptoms is significantly higher than in the general population. Particularly in genetic developmental and epileptic encephalopathies, epilepsy often coexists with autism spectrum disorders [113, 114]. These psychiatric comorbidities share multiple neurobiological mechanisms with epilepsy, including GABAergic signaling dysregulation, abnormal synaptic plasticity, disruption of functional connectivity, and neuroinflammatory responses [5]. For instance, dysfunction of parvalbumin‐positive inhibitory interneurons not only contributes to epileptogenesis but may also promote the emergence of psychiatric symptoms [115]. Thus, the pathological mechanisms of epilepsy‐related psychiatric disorders involve multiple dimensions, such as alterations in brain structure and function, neurotransmitter imbalances, neuroinflammatory mediators, and genetic factors.

Beyond neuropsychiatric comorbidities, epilepsy can also trigger systemic complications, such as neurogenic pulmonary edema. This condition may be attributed to massive catecholamine release, pulmonary vascular remodeling, and alveolar cell apoptosis [116]. Additionally, an association exists between epilepsy and stress cardiomyopathy. These conditions share overlapping genetic expression and molecular pathways, including the complement and coagulation cascades, as well as the HIF‐1 signaling pathway. This suggests that neurological activity can directly affect cardiac function [117]. Together, these findings indicate that effective epilepsy management requires approaches that extend beyond seizure control and address the broader neurological and systemic consequences of the disease.

### 3.3 Insufficient Precision in Diagnosis and Treatment

Epilepsy exhibits high clinical and etiological heterogeneity. The traditional “one‐size‐fits‐all” treatment approach shows limited efficacy for many patients, particularly those with genetic epilepsies. Achieving early diagnosis, accurate classification, and individualized precision therapy remains a major challenge in current clinical practice.

Numerous studies indicate that genetic heterogeneity is a key feature of epilepsy. More than 40 ion channel genes have been linked to epileptogenesis, with complex relationships among genotype, functional alteration, and clinical phenotype [4]. For example, different SCN1A mutations can produce a spectrum of disorders ranging from Genetic Epilepsy with Febrile Seizures Plus (GEFS+) to severe Dravet syndrome [4, 118]. Similarly, variants in potassium channel genes such as KCNQ2 and KCNT1 are important causes of developmental and epileptic encephalopathies, and variant‐specific therapies, including retigabine and quinidine, have been explored with variable efficacy [5, 119, 120]. These findings highlight both the opportunities and challenges of precision medicine in epilepsy, where accurate molecular diagnosis is increasingly important for selecting appropriate therapeutic strategies.

Although electroencephalography (EEG) remains the cornerstone for epilepsy diagnosis, traditional visual analysis methods exhibit inherent subjectivity, which limits their precision. In contrast, deep learning and artificial intelligence technologies—such as models integrating convolutional neural networks with the multi‐head self‐attention mechanisms of Transformers—can enhance both the accuracy and generalization ability of EEG signal analysis, providing new opportunities for more precise diagnosis and classification of epilepsy [121]. In terms of treatment strategies, for drug‐resistant epilepsy, the key to successful surgery lies in the precise localization of the epileptogenic network. For neuromodulation therapies such as DBS—for example, stimulation of the anterior thalamic nucleus—variability in efficacy may be related to the strength of functional connectivity between the stimulated brain regions and functional networks like the default mode network [122]. Meanwhile, theoretical modeling studies also provide a mechanistic basis for the effects of electrical stimulation at different targets, such as the subthalamic nucleus [123, 124]. Together, these advances highlight a shift from syndrome‐based management toward mechanism‐informed precision medicine, integrating intelligent diagnostics, network‐guided interventions, and individualized therapeutic strategies.

### 3.4 Sudden Unexpected Death in Epilepsy (SUDEP)

Despite advances in antiseizure medications, neuromodulation, and precision medicine, Sudden Unexpected Death in Epilepsy (SUDEP) remains one of the most devastating complications of epilepsy and a leading cause of epilepsy‐related mortality, particularly among adults and patients with DRE such as Dravet syndrome [125, 126]. Although its exact mechanisms remain incompletely understood, SUDEP is generally attributed to central cardiopulmonary alterations triggered by seizures, especially generalized tonic–clonic seizures. These changes may lead to terminal apnea and cardiac arrest [127, 128]. Therefore, elucidating the underlying mechanisms is crucial for clinical prevention. Regarding cardiac mechanisms, SUDEP patients often exhibit reduced heart rate variability, prolonged QT intervals, and various arrhythmias. Studies show that epilepsy upregulates neuronal sodium channels in the heart, such as NaV1.1. This alters sodium current properties and enhances cellular excitability, ultimately leading to fatal arrhythmias [129]. Moreover, Kv1.1 potassium channel‐deficient mouse models display neurogenic heart block and bradycardia. This suggests that abnormally elevated parasympathetic tone may contribute to SUDEP [130]. Additionally, Takotsubo syndrome, or apical ballooning syndrome, can occasionally occur post‐seizure. This condition is also considered potentially linked to SUDEP [131]. Collectively, these findings highlight the critical role of cardiac abnormalities in SUDEP pathogenesis.

In terms of respiratory mechanisms, central and obstructive apnea are central to the process of SUDEP, with laryngospasm identified as a key mechanism. Animal studies indicate that seizures can trigger gastric acid reflux into the larynx, inducing laryngospasm and further leading to obstructive apnea or even death. In experimental models, blocking acid reflux or applying laryngospasm notch manipulation has been shown to effectively prevent or alleviate these outcomes [132, 133, 134]. Analysis of actual SUDEP cases also reveals that terminal central apnea often precedes cardiac arrest. Thus, respiratory dysfunction plays a critical role in the progression of SUDEP. Imbalanced neuromodulation is also involved in the pathophysiology of SUDEP. This involves functional abnormalities in neurotransmitter systems such as serotonin (5‐HT), adenosine, and orexin [127, 128]. These systems collectively regulate cardiovascular and respiratory centers in the brainstem. Their dysregulation may lead to failure in the timely recovery of post‐ictal cardiorespiratory homeostasis. Pharmacological interventions targeting these neuromodulatory systems, along with neurostimulation and epilepsy surgery, have been reported to potentially reduce long‐term SUDEP risk [128]. These findings highlight potential therapeutic avenues for future treatment strategies.

Understanding the mechanisms underlying SUDEP has important implications for risk reduction and prevention. Current strategies focus on optimizing seizure control, identifying high‐risk individuals, and improving peri‐ictal monitoring and intervention [133]. For patients with severe and drug‐resistant epilepsies, particularly those with Dravet syndrome, advances in precision medicine, neuromodulation, and mechanism‐based therapies may provide new opportunities to reduce long‐term SUDEP risk [126]. Integrating these approaches may help improve outcomes in populations at highest risk of epilepsy‐related mortality.

## 4 Potential Therapeutic Strategies and Future Directions

Although more than 30 ASMs are currently approved, about 30% of patients develop DRE, highlighting the need for more effective therapeutic strategies. These strategies include precision medicine, targeted therapy, drug repositioning, non‐pharmacological interventions, and early intervention (Figure 3 right). The therapeutic strategies discussed below are presented according to their current level of evidence, ranging from FDA‐approved therapies and clinical‐stage interventions to preclinical studies and exploratory drug‐repositioning or network‐pharmacology–based approaches (Table 1).

**TABLE 1:** Therapeutic strategies for epilepsy: mechanisms, clinical status, and levels of evidence.

Therapy	Target/Mechanism	Evidence level	Clinical status
Cannabidiol	Multi‐target modulation involving endocannabinoid signaling, TRPV1, and neuronal excitability	FDA‐approved	Dravet syndrome, Lennox–Gastaut syndrome, and TSC‐associated seizures
Fenfluramine	Serotonergic modulation	FDA‐approved	Dravet syndrome and Lennox–Gastaut syndrome
Everolimus	mTOR inhibition	FDA‐approved; clinical trial‐supported	Adjunctive therapy for TSC‐associated focal/partial‐onset seizures
Retigabine/Ezogabine	Kv7 channel opener	Previously approved	Withdrawn/discontinued from market
Quinidine	KCNT1 channel blockade	Limited clinical evidence	KCNT1‐related epilepsy; efficacy remains inconsistent
Natalizumab	Anti‐inflammatory therapy targeting leukocyte trafficking	Negative or limited clinical evidence	Investigational; not established for epilepsy treatment
ANT‐DBS	Network modulation via anterior thalamic stimulation	Approved; SANTE trial‐supported	Adjunctive therapy for drug‐resistant focal/partial‐onset epilepsy
VNS	Network and neuromodulatory regulation	Approved	Established adjunctive therapy for drug‐resistant epilepsy
Sitagliptin	DPP‐4/PKC‐γ‐related signaling	Preclinical	Animal studies
Quercetin	Multi‐target anti‐inflammatory and antioxidant effects	Preclinical	Experimental studies
Apigenin	mTOR‐related and anti‐inflammatory modulation	Preclinical	Experimental studies
Kaempferol	Calcium signaling and multi‐target modulation	Network‐pharmacology prediction	Exploratory; requires experimental and clinical validation

### 4.1 Precision Medicine and Targeted Therapy

Increasing evidence suggests that effective epilepsy management requires patient stratification based on clinical, molecular, and network‐level characteristics. Traditional classification relies on seizure semiology, electroencephalographic patterns, and neuroimaging findings; however, these approaches often fail to capture the biological heterogeneity of epilepsy. The growing use of targeted gene panels and whole‐exome sequencing has improved diagnostic accuracy, particularly in developmental and epileptic encephalopathies, enabling genotype‐informed therapeutic decisions. Examples include avoidance of sodium channel blockers in Dravet syndrome caused by SCN1A loss‐of‐function variants and pharmacogenetic screening for HLA‐B*1502 prior to carbamazepine treatment in susceptible populations. Beyond genetics, network‐based biomarkers derived from EEG and neuroimaging may further facilitate individualized treatment selection and prediction of therapeutic response. Together, these advances are driving the transition from syndrome‐based management toward precision medicine, in which therapies are increasingly tailored to the molecular and network characteristics of individual patients.

#### 4.1.1 Targeting Ion Channels

Ion channelopathies are among the most common causes of genetic epilepsy. Developing drugs that target specific channel functions has become a key direction in precision medicine. For potassium channels, loss‐of‐function mutations in KCNQ2/3 genes, which encode Kv7.2/7.3 subunits, can increase neuronal excitability. Besides the previously approved Kv7 channel opener retigabine, developing more effective Kv7 activators with fewer side effects is a current research focus [119, 135]. Meanwhile, gain‐of‐function mutations in KCNT1 are linked to early‐onset epileptic encephalopathy. Although quinidine has been used clinically, its efficacy remains debated and inconsistent across studies. Thus, developing highly selective KCNT1 blockers is a promising strategy [120]. Additionally, gain‐of‐function mutations in BK channels, such as D434G, can induce epilepsy and paroxysmal movement disorders. Preclinical studies indicate that its specific blocker, paxilline, effectively suppresses neuronal hyperexcitability and alleviates symptoms [136]. Recently, mutations in KCNC2‐encoded Kv3.2 channels, like p.Cys125Tyr, were found to disrupt neuronal excitation by stabilizing the open conformation. This suggests Kv3.2 as a novel target for DEE [137]. Together, these studies advance precision treatment strategies for different potassium channel subtypes.

In the realm of sodium channels, Dravet syndrome caused by SCN1A haploinsufficiency serves as a classic example. Multiple therapeutic strategies have now demonstrated potential. For instance, low‐dose clonazepam, fenfluramine, and the multitarget agent cannabidiol not only help control seizures but also ameliorate autism‐like symptoms. Among these, cannabidiol—with its multifaceted mechanisms involving anticonvulsant, neuroprotective, anti‐inflammatory, and antioxidant effects—has received FDA approval for treating both Dravet syndrome and Lennox–Gastaut syndrome [5, 138]. These advances highlight the critical importance of selecting individualized treatment strategies based on channel pathophysiology in the management of DRE.

#### 4.1.2 Regulation of Specific Pathways

Hyperactivation of the mTOR signaling pathway is a common pathological mechanism in tuberous sclerosis complex and some SCN1A‐related developmental and epileptic encephalopathies. Studies indicate that early intervention before the age of 3, along with the use of mTOR inhibitors such as everolimus (FDA‐approved for tuberous sclerosis complex‐associated epilepsy and supported by the EXIST‐3 trial), is critical for controlling seizures and related comorbidities [5]. Furthermore, the natural flavonoid apigenin has demonstrated significant antiepileptic effects in a kainic acid‐induced model by inhibiting mTOR activity (preclinical evidence), suggesting its potential value as an adjunct therapy [139]. In epilepsy associated with cortical malformations, targeted therapy against this pathway also shows broad prospects [140]. Therefore, targeting the mTOR pathway has become one of the major directions in current epilepsy treatment research.

Neuroinflammation is recognized as a key driving factor in the onset and progression of epilepsy. Accumulating evidence supports targeting key inflammatory mediators, such as the NLRP3 inflammasome and the complement receptor C5aR1. Moreover, modulating microglial polarization from the pro‐inflammatory M1 phenotype to the anti‐inflammatory M2 phenotype represents a promising disease‐modifying strategy [141, 142]. For instance, natalizumab, an anti‐α4 integrin monoclonal antibody approved for multiple sclerosis and Crohn's disease, has been explored as a potential anti‐inflammatory strategy for DRE [143]. These findings underscore the clinical value of neuroinflammatory pathways as therapeutic targets.

Epigenetic regulatory mechanisms, including histone modifications, DNA methylation, and non‐coding RNAs, are widely involved in the dysregulation of gene expression in epilepsy. The histone deacetylase inhibitor SAHA increases the acetylation levels of histone H3 and H4 in GABRG2 mutation models. This restores the expression of GABA receptor subunits and exerts anti‐epileptic effects (preclinical evidence) [144]. Conversely, studies have also identified extensive reorganization of cytoplasmic mRNA polyadenylation in temporal lobe epilepsy. Upregulation of CPEB4 may influence epileptogenesis by modulating the poly(A) tail length of target genes [145]. Thus, epigenetic targets offer promising new avenues for developing novel anti‐epileptic therapies.

### 4.2 Development of New Drugs and Drug Repositioning

#### 4.2.1 Natural Medicines and Plant Extracts

Natural medicines have garnered widespread attention for their anti‐epileptic effects due to their multi‐target and low‐toxicity characteristics. Network pharmacology and molecular docking techniques have been applied to explore the multi‐target mechanisms of various traditional Chinese medicines and plant extracts. Examples include Huanglian, Kangxian Decoction, Chaibeizhixian Decoction, Danshen Dripping Pills, Yuganzi, as well as the plants *Achyranthes aspera* and Colebrookea oppositifolia [109]. For instance, quercetin exhibits anti‐epileptic and neuroprotective effects in animal models (preclinical evidence). Its mechanisms involve antioxidant and anti‐inflammatory activities, along with modulation of GABAergic signaling [2]. Similarly, apigenin reduces neuronal death and mossy fiber sprouting by inhibiting the mTOR pathway (preclinical evidence) [139]. Additionally, kaempferol is predicted via network pharmacology to alleviate neuronal hyperexcitability and calcium ion cytotoxicity. This is achieved by regulating calcium signaling pathways and AMPAR activity (network‐pharmacology prediction with limited experimental validation) [146]. Moreover, resveratrol, a polyphenolic compound, has demonstrated anti‐epileptic, neuroprotective, and antidepressant effects in preclinical studies [110]. Kangxian Decoction exerts anti‐epileptic effects by modulating neurotransmitters and neuroinflammatory pathways, such as IL‐17, TNF, and NF‐κB signaling (preclinical evidence) [147]. Collectively, these studies reveal the multi‐level mechanisms of natural components in epilepsy treatment, providing a theoretical basis for developing novel ASMs.

#### 4.2.2 Drug Repositioning

Utilizing marketed drugs for epilepsy treatment can significantly shorten the drug development cycle and reduce costs, making drug repositioning an attractive therapeutic strategy. For instance, sitagliptin, a diabetes drug and DPP‐4 inhibitor, exhibits notable anti‐epileptic effects in a lamotrigine‐resistant pentylenetetrazol model (preclinical evidence only). Its mechanism involves inhibiting PKC‐γ, modulating oxidative stress and inflammatory responses, and influencing the long‐term potentiation pathway [148], providing experimental support for drug repurposing. On the other hand, ceftriaxone has been investigated as a potential SLC1A2‐modulating therapy to enhance astrocytic glutamate uptake and reduce extracellular glutamate levels. However, clinical evidence remains limited. In a reported patient with SLC1A2‐related epilepsy, ceftriaxone treatment did not result in a significant reduction in seizure burden, suggesting that its therapeutic efficacy in epilepsy remains uncertain and requires further clinical evaluation [7]. Further studies show that rufinamide corrects abnormalities in sodium channel inactivation kinetics in neurons from Dup15q syndrome patients, establishing a mechanistic rationale for its potential use in this genetic epilepsy syndrome [24] and highlighting the importance of precision therapy targeting specific molecular mechanisms. Additionally, levetiracetam, an established anti‐seizure medication, was initially developed as a nootropic agent but was later found to possess anti‐epileptic activity. In a cobalt‐induced chronic epilepsy model, late administration of 200 mg/kg significantly suppressed hippocampal and cortical epileptiform discharges (preclinical evidence), potentially through regulation of the Bax/Bcl‐2 ratio and apoptosis‐related pathways [149, 150], indicating that multi‐mechanistic actions help control seizures across different stages. These examples illustrate how drug repositioning may expand therapeutic opportunities, although clinical validation remains necessary for many candidate agents.

### 4.3 Non‐Pharmacological Interventions

For DRE, non‐pharmacological interventions are important treatment options. Neuroregulation techniques modulate neural network activity through electrical stimulation of specific brain regions. DBS targeting the anterior nucleus of the thalamus (ANT) has shown therapeutic potential. The efficacy of anterior thalamic nucleus DBS was established in the Stimulation of the Anterior Nucleus of the Thalamus for Epilepsy (SANTE) trial, which demonstrated sustained seizure reduction during long‐term follow‐up [151, 152]. Studies indicate that responders exhibit enhanced connectivity with the default mode network (DMN) and anticorrelated connectivity with the hippocampus. This may suppress seizures by raising the seizure propagation threshold and increasing hippocampal GABA concentrations. Additionally, computational models suggest that electrical stimulation of the subthalamic nucleus (STN) also has potential in suppressing epileptic seizures. Its mechanism may involve the modulation of neuronal network dynamics [123]. Vagus nerve stimulation (VNS) is the most widely used neuroregulation therapy. It not only reduces seizure frequency but also improves epilepsy‐related comorbidities such as depression. Its physiological basis, patient selection, and treatment outcomes have been extensively studied [153]. Expert consensus also emphasizes the key role of VNS in pediatric epilepsy treatment [154]. Clinical benefit, long‐term safety, and broad applicability of VNS have been supported by extensive clinical experience and contemporary evidence, including recent studies on patient selection, treatment outcomes, and expert consensus recommendations in pediatric epilepsy [153, 154]. Responsive neurostimulation (RNS) serves as a closed‐loop system that can intervene immediately upon detecting abnormal discharges, thereby preventing seizure development. The efficacy of RNS was demonstrated in the pivotal NeuroPace trial, with long‐term follow‐up studies showing progressive seizure reduction over time [155, 156]. Music therapy, such as listening to Mozart's Sonata K.448 (the “Mozart effect”), has been shown to reduce epileptic activity on EEG in patients with epilepsy. This effect may be related to the periodic repetition within the musical structure, offering new avenues for adjunctive therapy [157]. Together, these neuromodulation strategies provide important therapeutic options for patients with drug‐resistant epilepsy who are not suitable candidates for curative surgery.

For patients with clear epileptogenic foci, surgical resection remains a potentially curative approach. Both resective epilepsy surgery and neuromodulation surgery have been shown to positively reduce the long‐term risk of sudden unexpected death in epilepsy (SUDEP) [128]. Laser interstitial thermal therapy (LITT) is a minimally invasive surgical technique that is increasingly used to treat brain tumors and epilepsy, particularly for lesions located in deep or functional areas [158]. Large prospective multicenter studies have further confirmed that LITT is a well‐tolerated therapy for drug‐resistant mesial temporal lobe epilepsy, and it significantly improves seizure outcomes and quality of life [159]. Therefore, with precise localization of epileptogenic foci, surgical treatment offers the possibility of a cure for some patients.

The ketogenic diet is a high‐fat, very low‐carbohydrate dietary therapy. It has been used since the 1920s to treat DRE in children. Its anticonvulsant efficacy, safety, and tolerability are supported by extensive clinical and scientific evidence [160]. In recent years, its application in pediatric epilepsy has continued to expand. This includes in‐depth research on its mechanisms of action, the introduction of alternatives such as the modified Atkins diet, and broader promotion across different age groups and regions [160]. Studies show that the ketogenic diet confers antiepileptic effects in mice by altering the composition and function of the gut microbiome. The associated metabolic features involve anaplerosis, fatty acid β‐oxidation, and amino acid metabolism [161]. Optimizing the ketogenic diet treatment regimen and identifying key factors for seizure control and psychomotor enhancement are crucial. These efforts are essential for improving therapeutic outcomes in children with drug‐resistant epilepsy [162]. Therefore, metabolic regulation therapy plays an indispensable role in the comprehensive management of DRE. Beyond neuromodulation and dietary interventions, psychological approaches such as cognitive behavioral therapy may improve quality of life, alleviate depressive symptoms, and enhance treatment adherence in patients with epilepsy, thereby contributing to comprehensive long‐term disease management.

### 4.4 Early Intervention and Disease Modification

Intervening during the “silent phase” of epileptogenesis represents an ideal therapeutic strategy to prevent, reverse, or halt the development of chronic epilepsy. Achieving this goal requires accurate identification of reliable biomarkers, along with early modulation of key pathological processes such as neuroinflammation, autophagy, and epigenetic regulation [96]. Therefore, early identification and intervention are critical for improving epilepsy prognosis.

Biomarkers include imaging features, electroencephalogram (EEG) patterns, and various molecular markers. High‐frequency oscillations have emerged as promising EEG biomarkers of epileptogenic networks, whereas blood‐ and cerebrospinal fluid‐based markers such as glial fibrillary acidic protein (GFAP) and neurofilament light chain (NfL) may facilitate disease monitoring. In addition, peripheral DNA methylation signatures and miRNAs are being investigated as minimally invasive biomarkers for early diagnosis and prognosis. For instance, serum miR‐4465 demonstrates potential as a diagnostic biomarker for epilepsy by targeting PTEN‐mediated astrocyte autophagy [163]. These biomarkers may not only facilitate early diagnosis and disease monitoring but also improve patient stratification for precision therapeutic interventions.

In post‐traumatic epilepsy (PTE), neuroinflammation and autophagy are closely interconnected, evidenced by abnormal microglial polarization and dysfunctional autophagy. Early administration of anti‐inflammatory drugs, such as C5aR1 antagonists, or autophagy modulators may thereby alter the natural disease course [142]. This implies that combined interventions targeting neuroinflammation and autophagy could yield synergistic therapeutic effects. Epigenetic regulation also plays a pivotal role in epileptogenesis, involving mechanisms like histone modifications and DNA methylation. Early use of histone deacetylase inhibitors (HDACi) may suppress epileptogenesis by rebalancing the expression of related genes [3]. Thus, epigenetics‐targeted therapy provides novel insights for epilepsy prevention and management.

Early biomarker identification combined with integrated regulation of neuroinflammation, autophagy, and epigenetic mechanisms may help block epileptogenesis and improve long‐term outcomes. Importantly, these disease‐modifying strategies should be distinguished from conventional anti‐seizure therapies. Whereas current ASMs primarily suppress seizure occurrence, disease‐modifying interventions aim to prevent or delay epileptogenesis and alter long‐term disease progression. Evidence from mTOR inhibition in tuberous sclerosis complex‐associated epilepsy, particularly the clinical benefits observed with everolimus, together with emerging anti‐inflammatory approaches suggests that early intervention during critical windows may offer opportunities to modify disease trajectory rather than simply control symptoms.

However, the successful implementation of disease‐modifying strategies will require reliable biomarkers for patient selection and treatment monitoring, together with improved understanding of the temporal window during which epileptogenic processes remain reversible. Additional challenges include long‐term safety evaluation, biomarker validation across heterogeneous patient populations, and the translation of promising preclinical findings into effective clinical therapies. Addressing these barriers will be essential for advancing precision medicine and disease‐modifying interventions in epilepsy.

## 5 Summary and Outlook

Epilepsy is a complex chronic brain network disorder whose pathological mechanisms extend far beyond the traditional “channelopathy” concept. It arises from a multidimensional network encompassing genetic variations, neurotransmitter imbalances, neuroinflammation, disrupted cellular homeostasis, epigenetic dysregulation, and metabolic‐mitochondrial dysfunction. While ion channel mutations directly alter neuronal excitability, non‐ion channel genes contribute through diverse mechanisms such as disrupted cell adhesion and metabolism. Concurrently, neuroinflammation, dysregulated autophagy, apoptosis, and ferroptosis, epigenetic modifications, and mitochondrial stress collectively drive disease progression. Despite these insights, about one‐third of patients develop drug‐resistant epilepsy (DRE), mediated by altered drug targets, overexpression of efflux transporters, and pathological network remodeling. Drug side effects, neuropsychiatric comorbidities, and SUDEP further underscore the limitations of conventional, symptom‐focused therapies. This understanding is shifting treatment paradigms from seizure control toward disease modification.

Major challenges remain, including limited translation of preclinical findings, biological heterogeneity among patients, inadequate patient stratification, difficulties in biomarker validation, and regulatory barriers for emerging gene‐based therapies. To address these challenges, future strategies emphasize precision, multi‐target, and early interventions. Precision medicine leads the way with therapies targeting specific gene mutations (e.g., KCNQ2, SCN1A) and key pathways (e.g., mTOR, NLRP3). Advances in genetic testing, network‐based biomarkers, and circulating molecular biomarkers may further facilitate individualized treatment selection and improve clinical decision‐making. Epigenetic drugs, natural multi‐target compounds, and drug repurposing offer complementary avenues. Non‐pharmacological approaches—including neuromodulation and the ketogenic diet—are crucial for DRE. Importantly, early intervention during the “silent period,” guided by biomarkers and targeting processes like neuroinflammation and autophagy, holds promise for altering disease trajectory. Ultimately, overcoming epilepsy requires integrating neuroscience, genetics, immunology, and clinical medicine into a holistic precision framework—from genetic diagnosis and network modulation to early biomarker detection and individualized therapy. Multidisciplinary collaboration and technological innovation will further advance this framework, facilitating the transition from seizure suppression toward precision, preventive, and disease‐modifying epilepsy care.

## Author Contributions

Y.H., J.X. and Y.S. wrote the main manuscript text, and J.Z., X.L. and X.Y. prepared Figures 1, 2, 3. L.Q. and H.S. supervised the project, acquired funding, and provided critical revisions. All authors reviewed and approved the final version of the manuscript.

## Funding

This work was funded by the National Natural Science Foundation of China (Nos. 32130060, 82401633, and 82072160), the Basic Research Program of Jiangsu (No. BK20232023), the Natural Science Research Projects in Universities of Jiangsu Province (Nos. 24KJA310007 and 24KJB310013), and the Nantong Science and Technology Program (No. 20241118084).

## Conflicts of Interest

The authors declare no conflicts of interest.

## Data Availability

The data that support the findings of this study are available from the corresponding author upon reasonable request.

## References

[1] J. Chen , Y. Gao , N. Liu , et al., “Mechanism of NLRP3 Inflammasome in Epilepsy and Related Therapeutic Agents,” Neuroscience 546 (2024): 157–177. 10.1016/j.neuroscience.2024.03.029 38574797

[2] E. Akyuz , Y. N. Paudel , A. K. Polat , H. E. Dundar , and E. Angelopoulou , “Enlightening the Neuroprotective Effect of Quercetin in Epilepsy: From Mechanism to Therapeutic Opportunities,” Epilepsy & Behavior 115 (2021): 107701. 10.1016/j.yebeh.2020.107701 33412369

[3] S. Chen , M. Huang , D. Xu , and M. Li , “Epigenetic Regulation in Epilepsy: A Novel Mechanism and Therapeutic Strategy for Epilepsy,” Neurochemistry International 173 (2024): 105657. 10.1016/j.neuint.2023.105657 38145842

[4] F. Wei , L. M. Yan , T. Su , et al., “Ion Channel Genes and Epilepsy: Functional Alteration, Pathogenic Potential, and Mechanism of Epilepsy,” Neuroscience Bulletin 33, no. 4 (2017): 455–477. 10.1007/s12264-017-0134-1 PMC5567559 28488083

[5] N. Specchio , V. Di Micco , E. Aronica , et al., “The Epilepsy‐Autism Phenotype Associated With Developmental and Epileptic Encephalopathies: New Mechanism‐Based Therapeutic Options,” Epilepsia 66, no. 4 (2025): 970–987. 10.1111/epi.18209 39985505

[6] C. C. Homan , S. Pederson , T. H. To , et al., “PCDH19 Regulation of Neural Progenitor Cell Differentiation Suggests Asynchrony of Neurogenesis as a Mechanism Contributing to PCDH19 Girls Clustering Epilepsy,” Neurobiology of Disease 116 (2018): 106–119. 10.1016/j.nbd.2018.05.004 29763708

[7] A. B. Stergachis , J. Pujol‐Giménez , G. Gyimesi , et al., “Recurrent SLC1A2 Variants Cause Epilepsy via a Dominant Negative Mechanism,” Annals of Neurology 85, no. 6 (2019): 921–926. 10.1002/ana.25477 PMC6800210 30937933

[8] A. Vega García , M. L. López‐Meraz , M. I. González , L. Rocha , J. E. Peixoto‐Santos , and E. A. Cavalheiro , “Immunity and Neuroinflammation in Early Stages of Life and Epilepsy,” Epilepsia 66, no. 7 (2025): 2157–2169. 10.1111/epi.18361 PMC12291034 40072465

[9] K. M. J. Van Loo , G. L. Carvill , A. J. Becker , et al., “Epigenetic Genes and Epilepsy—Emerging Mechanisms and Clinical Applications,” Nature Reviews. Neurology 18, no. 9 (2022): 530–543. 10.1038/s41582-022-00693-y 35859062

[10] N. M. Beckonert , T. Opitz , J. Pitsch , P. Soares da Silva , and H. Beck , “Polyamine Modulation of Anticonvulsant Drug Response: A Potential Mechanism Contributing to Pharmacoresistance in Chronic Epilepsy,” Journal of Neuroscience 38, no. 24 (2018): 5596–5605. 10.1523/JNEUROSCI.0640-18.2018 PMC8174136 29789377

[11] J. Xiong , D. A. Mao , and L. Q. Liu , “Research Progress on the Role of ABC Transporters in the Drug Resistance Mechanism of Intractable Epilepsy,” BioMed Research International 2015 (2015): 194541. 10.1155/2015/194541 PMC4600483 26491660

[12] W. Woldman , M. J. Cook , and J. R. Terry , “Evolving Dynamic Networks: An Underlying Mechanism of Drug Resistance in Epilepsy?,” Epilepsy & Behavior 94 (2019): 264–268. 10.1016/j.yebeh.2019.03.003 PMC6581121 30981121

[13] S. Jiang , H. Li , L. Zhang , et al., “Generic Diagramming Platform (GDP): A Comprehensive Database of High‐Quality Biomedical Graphics,” Nucleic Acids Research 53, no. D1 (2025): D1670–D1676. 10.1093/nar/gkae973 PMC11701665 39470721

[14] V. Lolam and A. Roy , “Developmental Mechanisms Underlying Pediatric Epilepsy,” Frontiers in Neurology 16 (2025): 1586947. 10.3389/fneur.2025.1586947 PMC12172511 40529445

[15] Y. Tian , Y. Q. Hou , Q. X. Zhai , et al., “RYR3 Variants Are Potentially Associated With Idiopathic (Non‐Lesional) Partial Epilepsy/Susceptibility of Seizures, Toward Understanding the Gene‐Disease Association by Genetic Dependent Nature,” American Journal of Medical Genetics. Part B, Neuropsychiatric Genetics 198, no. 5 (2025): e33023. 10.1002/ajmg.b.33023 39840699

[16] Y. Yang , Q. Zeng , M. Cheng , et al., “GABRB3‐Related Epilepsy: Novel Variants, Clinical Features and Therapeutic Implications,” Journal of Neurology 269, no. 5 (2022): 2649–2665. 10.1007/s00415-021-10834-w 34698933

[17] F. Paduano , E. Colao , S. Loddo , et al., “7q35 Microdeletion and 15q13.3 and Xp22.33 Microduplications in a Patient With Severe Myoclonic Epilepsy, Microcephaly, Dysmorphisms, Severe Psychomotor Delay and Intellectual Disability,” Genes (Basel) 11, no. 5 (2020): 525. 10.3390/genes11050525 PMC7288449 32397165

[18] A. M. Tidball , L. F. Lopez‐Santiago , Y. Yuan , et al., “Variant‐Specific Changes in Persistent or Resurgent Sodium Current in SCN8A‐Related Epilepsy Patient‐Derived Neurons,” Brain 143, no. 10 (2020): 3025–3040. 10.1093/brain/awaa247 PMC7780473 32968789

[19] C. Bar , M. Kuchenbuch , G. Barcia , et al., “Developmental and Epilepsy Spectrum of KCNB1 Encephalopathy With Long‐Term Outcome,” Epilepsia 61, no. 11 (2020): 2461–2473. 10.1111/epi.16679 32954514

[20] Q. L. Miao , S. Herlitze , M. D. Mark , and J. L. Noebels , “Adult Loss of Cacna1a in Mice Recapitulates Childhood Absence Epilepsy by Distinct Thalamic Bursting Mechanisms,” Brain 143, no. 1 (2020): 161–174. 10.1093/brain/awz365 PMC6935748 31800012

[21] H. Tao , Z. Chen , J. Wu , et al., “DNA Methylation Signature of Epileptic Encephalopathy‐Related Pathogenic Genes Encoding Ion Channels in Temporal Lobe Epilepsy,” Frontiers in Neurology 12 (2021): 692412. 10.3389/fneur.2021.692412 PMC8358672 34393975

[22] H. Ishiura , K. Doi , J. Mitsui , et al., “Expansions of Intronic TTTCA and TTTTA Repeats in Benign Adult Familial Myoclonic Epilepsy,” Nature Genetics 50, no. 4 (2018): 581–590. 10.1038/s41588-018-0067-2 29507423

[23] E. J. Romasko , E. T. DeChene , J. Balciuniene , et al., “PCDH19‐Related Epilepsy in a Male With Klinefelter Syndrome: Additional Evidence Supporting PCDH19 Cellular Interference Disease Mechanism,” Epilepsy Research 145 (2018): 89–92. 10.1016/j.eplepsyres.2018.06.008 29933145

[24] M. Elamin , F. Lemtiri‐Chlieh , T. M. Robinson , and E. S. Levine , “Dysfunctional Sodium Channel Kinetics as a Novel Epilepsy Mechanism in Chromosome 15q11‐q13 Duplication Syndrome,” Epilepsia 64, no. 9 (2023): 2515–2527. 10.1111/epi.17687 PMC10529833 37329181

[25] N. He , B. Z. Guan , J. Wang , et al., “HCFC1 Variants in the Proteolysis Domain Are Associated With X‐Linked Idiopathic Partial Epilepsy: Exploring the Underlying Mechanism,” Clinical and Translational Medicine 13, no. 6 (2023): e1289. 10.1002/ctm2.1289 PMC10235798 37264743

[26] X. G. Ye , Z. G. Liu , J. Wang , et al., “YWHAG Mutations Cause Childhood Myoclonic Epilepsy and Febrile Seizures: Molecular Sub‐Regional Effect and Mechanism,” Frontiers in Genetics 12 (2021): 632466. 10.3389/fgene.2021.632466 PMC7985244 33767733

[27] J. H. Zhu , Z. Chen , C. F. Cheng , et al., “ATP2B1 Variants Associated With Generalized Epilepsy Without Neurodevelopmental Disorders and the Underlying Mechanism,” Seizure 131 (2025): 369–377. 10.1016/j.seizure.2025.08.017 40834682

[28] E. Akyuz , A. K. Polat , E. Eroglu , I. Kullu , E. Angelopoulou , and Y. N. Paudel , “Revisiting the Role of Neurotransmitters in Epilepsy: An Updated Review,” Life Sciences 265 (2021): 118826. 10.1016/j.lfs.2020.118826 33259863

[29] R. Mao , M. Hu , X. Liu , et al., “Impairments of GABAergic Transmission in Hippocampus Mediate Increased Susceptibility of Epilepsy in the Early Stage of Alzheimer's Disease,” Cell Communication and Signaling: CCS 22, no. 1 (2024): 147. 10.1186/s12964-024-01528-7 PMC10885444 38388921

[30] H. Y. Kim , P. G. Suh , and J. I. Kim , “The Role of Phospholipase C in GABAergic Inhibition and Its Relevance to Epilepsy,” International Journal of Molecular Sciences 22, no. 6 (2021): 3149. 10.3390/ijms22063149 PMC8003358 33808762

[31] Y. Wang , B. Tan , Y. Wang , and Z. Chen , “Cholinergic Signaling, Neural Excitability, and Epilepsy,” Molecules 26, no. 8 (2021): 2258. 10.3390/molecules26082258 PMC8070422 33924731

[32] L. Yang , Y. Wang , and Z. Chen , “Central Histaminergic Signalling, Neural Excitability and Epilepsy,” British Journal of Pharmacology 179, no. 1 (2022): 3–22. 10.1111/bph.15692 34599508

[33] M. A. Núñez‐Ochoa , G. A. Chiprés‐Tinajero , and L. Medina‐Ceja , “Evaluation of the Hippocampal Immunoreactivity of the Serotonin 5‐HT1A, 5‐HT2 and 5‐HT7 Receptors in a Pilocarpine Temporal Lobe Epilepsy Rat Model With Fast Ripples,” Neuroreport 32, no. 4 (2021): 306–311. 10.1097/WNR.0000000000001594 33470771

[34] D. Zarcone and S. Corbetta , “Shared Mechanisms of Epilepsy, Migraine and Affective Disorders,” Neurological Sciences 38, no. Suppl 1 (2017): 73–76. 10.1007/s10072-017-2902-0 28527083

[35] F. M. Werner and R. Coveñas , “Classical Neurotransmitters and Neuropeptides Involved in Generalized Epilepsy in a Multi‐Neurotransmitter System: How to Improve the Antiepileptic Effect?,” Epilepsy & Behavior 71, no. Pt B (2017): 124–129. 10.1016/j.yebeh.2015.01.038 25819950

[36] E. Akyuz , Z. Doganyigit , Y. N. Paudel , et al., “Immunoreactivity of Muscarinic Acetylcholine M2 and Serotonin 5‐HT2B Receptors, Norepinephrine Transporter and Kir Channels in a Model of Epilepsy,” Life (Basel) 11, no. 4 (2021): 276. 10.3390/life11040276 PMC8066555 33810231

[37] P. Solanki and S. Jha , “Innate Immune Activation and Neuroinflammatory Pathways in Epilepsy,” Cytokine & Growth Factor Reviews 84 (2025): 35–46. 10.1016/j.cytogfr.2025.06.009 40610341

[38] P. Nobili , W. Shen , K. Milicevic , J. Bogdanovic Pristov , E. Audinat , and L. Nikolic , “Therapeutic Potential of Astrocyte Purinergic Signalling in Epilepsy and Multiple Sclerosis,” Frontiers in Pharmacology 13 (2022): 900337. 10.3389/fphar.2022.900337 PMC9109958 35586058

[39] X. Dong , J. Fan , D. Lin , et al., “Captopril Alleviates Epilepsy and Cognitive Impairment by Attenuation of C3‐Mediated Inflammation and Synaptic Phagocytosis,” Journal of Neuroinflammation 19, no. 1 (2022): 226. 10.1186/s12974-022-02587-8 PMC9476304 36104755

[40] X. Mu , X. Zhang , H. Gao , L. Gao , Q. Li , and C. Zhao , “Crosstalk Between Peripheral and the Brain‐Resident Immune Components in Epilepsy,” Journal of Integrative Neuroscience 21, no. 1 (2022): 9. 10.31083/j.jin2101009 35164445

[41] G. Yamanaka , S. Morichi , T. Takamatsu , et al., “Links Between Immune Cells From the Periphery and the Brain in the Pathogenesis of Epilepsy: A Narrative Review,” International Journal of Molecular Sciences 22, no. 9 (2021): 4395. 10.3390/ijms22094395 PMC8122797 33922369

[42] L. Langenbruch , L. Bleß , A. Schulte‐Mecklenbeck , et al., “Blood and Cerebrospinal Fluid Immune Cell Profiles in Patients With Temporal Lobe Epilepsy of Different Etiologies,” Epilepsia 61, no. 10 (2020): e153–e158. 10.1111/epi.16688 32893887

[43] Y. W. Shin , S. B. Hong , Y. W. Shin , et al., “Systemic T Cell Receptor Profiling Reveals Adaptive Immune Activation and Potential Immune Signatures of Diagnosis and Brain Atrophy in Epilepsy,” Annals of Clinical and Translational Neurology 13 (2025): 296–309. 10.1002/acn3.70203 PMC12883695 41058231

[44] J. An , H. Li , D. Xia , et al., “The Role of Interleukin‐17 in Epilepsy,” Epilepsy Research 186 (2022): 107001. 10.1016/j.eplepsyres.2022.107001 35994860

[45] M. Chen , S. R. Edwards , and D. C. Reutens , “Complement in the Development of Post‐Traumatic Epilepsy: Prospects for Drug Repurposing,” Journal of Neurotrauma 37, no. 5 (2020): 692–705. 10.1089/neu.2019.6942 32000582

[46] X. Ping , W. Xiong , R. Priya , et al., “Blocking the Innate Immune CD1d/NKT Cell Axis Prevents the Development of Cortical Hyperexcitability and Posttraumatic Epilepsy,” Epilepsia 66, no. 6 (2025): 2110–2123. 10.1111/epi.18332 PMC12169402 40019476

[47] D. Cukovic , S. Bagla , D. Ukasik , et al., “Exosomes in Epilepsy of Tuberous Sclerosis Complex: Carriers of Pro‐Inflammatory MicroRNAs,” Noncoding RNA 7, no. 3 (2021): 40. 10.3390/ncrna7030040 PMC8293460 34287356

[48] X. Li , Q. Guo , Z. Zheng , X. Wang , and S. Liu , “Immune‐Mediated Epilepsy With GAD65 Antibodies,” Journal of Neuroimmunology 341 (2020): 577189. 10.1016/j.jneuroim.2020.577189 32087461

[49] A. R. Tröscher , K. M. Mair , L. Verdú de Juan , et al., “Temporal Lobe Epilepsy With GAD Antibodies: Neurons Killed by T Cells Not by Complement Membrane Attack Complex,” Brain 146, no. 4 (2023): 1436–1452. 10.1093/brain/awac404 PMC10115353 36314080

[50] J. Föhr , J. K. Prümmer , A. Maiolini , et al., “Cerebrospinal Fluid‐Specific Oligoclonal Bands in Dogs With Idiopathic Epilepsy,” Journal of Veterinary Internal Medicine 39, no. 1 (2025): e17265. 10.1111/jvim.17265 PMC11666169 39715535

[51] D. Caputo , R. Iorio , F. Vigevano , and L. Fusco , “Febrile Infection‐Related Epilepsy Syndrome (FIRES) With Super‐Refractory Status Epilepticus Revealing Autoimmune Encephalitis due to GABA(A)R Antibodies,” European Journal of Paediatric Neurology 22, no. 1 (2018): 182–185. 10.1016/j.ejpn.2017.11.005 29203057

[52] H. Zhu , W. Wang , and Y. Li , “Molecular Mechanism and Regulation of Autophagy and Its Potential Role in Epilepsy,” Cells 11, no. 17 (2022): 2621. 10.3390/cells11172621 PMC9455075 36078029

[53] Q. Li , Y. Han , J. Du , et al., “Alterations of Apoptosis and Autophagy in Developing Brain of Rats With Epilepsy: Changes in LC3, P62, Beclin‐1 and Bcl‐2 Levels,” Neuroscience Research 130 (2018): 47–55. 10.1016/j.neures.2017.08.004 28807642

[54] K. T. Sumadewi , S. Harkitasari , and D. C. Tjandra , “Biomolecular Mechanisms of Epileptic Seizures and Epilepsy: A Review,” Acta Epileptologica 5, no. 1 (2023): 28. 10.1186/s42494-023-00137-0 PMC11960269 40217521

[55] J. Gan , Y. Qu , J. Li , F. Zhao , and D. Mu , “An Evaluation of the Links Between microRNA, Autophagy, and Epilepsy,” Reviews in the Neurosciences 26, no. 2 (2015): 225–237. 10.1515/revneuro-2014-0062 25719305

[56] C. Romá‐Mateo , C. Aguado , J. L. García‐Giménez , E. Knecht , P. Sanz , and F. V. Pallardó , “Oxidative Stress, a New Hallmark in the Pathophysiology of Lafora Progressive Myoclonus Epilepsy,” Free Radical Biology & Medicine 88, no. Pt A (2015): 30–41. 10.1016/j.freeradbiomed.2015.01.034 25680286

[57] M. Lahuerta , D. Gonzalez , C. Aguado , et al., “Reactive Glia‐Derived Neuroinflammation: A Novel Hallmark in Lafora Progressive Myoclonus Epilepsy That Progresses With Age,” Molecular Neurobiology 57, no. 3 (2020): 1607–1621. 10.1007/s12035-019-01842-z PMC7062575 31808062

[58] X. Jiang , Y. Sui , J. Zhang , T. Yi , Y. Zhao , and X. Liu , “Advance of Research on Endoplasmic Reticulum Stress and Genetic Epilepsy,” Zhonghua Yi Xue Yi Chuan Xue Za Zhi 40, no. 6 (2023): 756–761. 10.3760/cma.j.cn511374-20221027-00724 37212016

[59] Z. He , S. Liu , W. Shi , et al., “Single‐Cell Transcriptome Analysis Reveals Dysregulation of Microglial Iron Homeostasis in Temporal Lobe Epilepsy,” Brain Research 1865 (2025): 149885. 10.1016/j.brainres.2025.149885 40803604

[60] D. Shen , Z. Zhao , Y. Ji , et al., “The Role of Ferroptosis in Epilepsy: Mechanisms and Targeted Therapies,” Biochemical Pharmacology 249 (2026): 117912. 10.1016/j.bcp.2026.117912 41865900

[61] M. Putra , S. S. Vasanthi , N. S. Rao , et al., “Inhibiting Inducible Nitric Oxide Synthase With 1400W Reduces Soman (GD)‐Induced Ferroptosis in Long‐Term Epilepsy‐Associated Neuropathology: Structural and Functional Magnetic Resonance Imaging Correlations With Neurobehavior and Brain Pathology,” Journal of Pharmacology and Experimental Therapeutics 388, no. 2 (2024): 724–738. 10.1124/jpet.123.001929 PMC10801728 38129129

[62] Q. Zan , L. Fan , R. Wang , et al., “Dual‐Channel Fluorescent Probe for Simultaneously Visualizing ONOO(−) and Viscosity in Epilepsy, Non‐Alcoholic Fatty Liver and Tumoral Ferroptosis Models,” Biosensors & Bioelectronics 282 (2025): 117495. 10.1016/j.bios.2025.117495 40273776

[63] S. Singh , T. G. Singh , A. K. Rehni , V. Sharma , M. Singh , and R. Kaur , “Reviving Mitochondrial Bioenergetics: A Relevant Approach in Epilepsy,” Mitochondrion 58 (2021): 213–226. 10.1016/j.mito.2021.03.009 33775871

[64] K. Zhang , Z. Yang , Z. Yang , et al., “Targeting Microglial GLP1R in Epilepsy: A Novel Approach to Modulate Neuroinflammation and Neuronal Apoptosis,” European Journal of Pharmacology 981 (2024): 176903. 10.1016/j.ejphar.2024.176903 39154823

[65] K. Conboy , D. C. Henshall , and G. P. Brennan , “Epigenetic Principles Underlying Epileptogenesis and Epilepsy Syndromes,” Neurobiology of Disease 148 (2021): 105179. 10.1016/j.nbd.2020.105179 33181318

[66] R. Citraro , A. Leo , M. Santoro , G. D'Agostino , A. Constanti , and E. Russo , “Role of Histone Deacetylases (HDACs) in Epilepsy and Epileptogenesis,” Current Pharmaceutical Design 23, no. 37 (2017): 5546–5562. 10.2174/1381612823666171024130001 29076408

[67] R. Jagirdar , M. Drexel , E. Kirchmair , R. O. Tasan , and G. Sperk , “Rapid Changes in Expression of Class I and IV Histone Deacetylases During Epileptogenesis in Mouse Models of Temporal Lobe Epilepsy,” Experimental Neurology 273 (2015): 92–104. 10.1016/j.expneurol.2015.07.026 26238735

[68] S. F. Miller‐Delaney , K. Bryan , S. Das , et al., “Differential DNA Methylation Profiles of Coding and Non‐Coding Genes Define Hippocampal Sclerosis in Human Temporal Lobe Epilepsy,” Brain 138, no. Pt 3 (2015): 616–631. 10.1093/brain/awu373 PMC4408428 25552301

[69] K. Kobow and I. Blümcke , “Epigenetic Mechanisms in Epilepsy,” Progress in Brain Research 213 (2014): 279–316. 10.1016/B978-0-444-63326-2.00014-4 25194494

[70] K. J. Dębski , A. Pitkanen , N. Puhakka , et al., “Etiology Matters ‐ Genomic DNA Methylation Patterns in Three Rat Models of Acquired Epilepsy,” Scientific Reports 6 (2016): 25668. 10.1038/srep25668 PMC4860710 27157830

[71] K. Kobow , I. Jeske , M. Hildebrandt , et al., “Increased Reelin Promoter Methylation Is Associated With Granule Cell Dispersion in Human Temporal Lobe Epilepsy,” Journal of Neuropathology and Experimental Neurology 68, no. 4 (2009): 356–364. 10.1097/NEN.0b013e31819ba737 19287316

[72] Q. Zhu , L. Wang , Y. Zhang , et al., “Increased Expression of DNA Methyltransferase 1 and 3a in Human Temporal Lobe Epilepsy,” Journal of Molecular Neuroscience 46, no. 2 (2012): 420–426. 10.1007/s12031-011-9602-7 21826395

[73] H. Y. Long , L. Feng , J. Kang , et al., “Blood DNA Methylation Pattern Is Altered in Mesial Temporal Lobe Epilepsy,” Scientific Reports 7 (2017): 43810. 10.1038/srep43810 PMC5343463 28276448

[74] R. Martins‐Ferreira , B. Leal , J. Chaves , et al., “Circulating Cell‐Free DNA Methylation Mirrors Alterations in Cerebral Patterns in Epilepsy,” Clinical Epigenetics 14, no. 1 (2022): 188. 10.1186/s13148-022-01416-2 PMC9795776 36575526

[75] G. P. Brennan and D. C. Henshall , “microRNAs in the Pathophysiology of Epilepsy,” Neuroscience Letters 667 (2018): 47–52. 10.1016/j.neulet.2017.01.017 28104433

[76] D. C. Henshall , H. M. Hamer , R. J. Pasterkamp , et al., “MicroRNAs in Epilepsy: Pathophysiology and Clinical Utility,” Lancet Neurology 15, no. 13 (2016): 1368–1376. 10.1016/S1474-4422(16)30246-0 27839653

[77] M. T. Venø , C. R. Reschke , G. Morris , et al., “A Systems Approach Delivers a Functional microRNA Catalog and Expanded Targets for Seizure Suppression in Temporal Lobe Epilepsy,” Proceedings of the National Academy of Sciences of the United States of America 117, no. 27 (2020): 15977–15988. 10.1073/pnas.1919313117 PMC7355001 32581127

[78] W. J. Lee , J. Moon , D. Jeon , et al., “Possible Epigenetic Regulatory Effect of Dysregulated Circular RNAs in Epilepsy,” PLoS One 13, no. 12 (2018): e0209829. 10.1371/journal.pone.0209829 PMC6310357 30592747

[79] C. Villa , M. Lavitrano , and R. Combi , “Long Non‐Coding RNAs and Related Molecular Pathways in the Pathogenesis of Epilepsy,” International Journal of Molecular Sciences 20, no. 19 (2019): 4898. 10.3390/ijms20194898 PMC6801574 31581735

[80] J. Mathoux , M. M. Wilson , S. Srinivas , et al., “N6‐Methyladenosine (m6A) Dysregulation Contributes to Network Excitability in Temporal Lobe Epilepsy,” JCI Insight 10, no. 14 (2025): e188612. 10.1172/jci.insight.188612 PMC12288969 40693462

[81] M. Xiao , X. Wang , E. Xiao , and Q. Ming , “Overexpression of FTO Alleviates Traumatic Brain Injury Induced Posttraumatic Epilepsy by Upregulating NR4A2 Expression via m6A Demethylation,” Functional & Integrative Genomics 25, no. 1 (2025): 17. 10.1007/s10142-024-01522-9 39825968

[82] I. Younus and D. S. Reddy , “Epigenetic Interventions for Epileptogenesis: A New Frontier for Curing Epilepsy,” Pharmacology & Therapeutics 177 (2017): 108–122. 10.1016/j.pharmthera.2017.03.002 PMC5565684 28279785

[83] A. Roopra , R. Dingledine , and J. Hsieh , “Epigenetics and Epilepsy,” Epilepsia 53, no. Suppl 9 (2012): 2–10. 10.1111/epi.12030 PMC3531878 23216574

[84] X. Zhang , Z. Wu , X. Zhou , and H. Tao , “Mitochondrial Dysfunction in Epilepsy: Mechanistic Insights and Clinical Strategies,” Molecular Biology Reports 52, no. 1 (2025): 470. 10.1007/s11033-025-10577-1 40392243

[85] J. R. Tenney , L. Rozhkov , P. Horn , L. Miles , and M. V. Miles , “Cerebral Glucose Hypometabolism Is Associated With Mitochondrial Dysfunction in Patients With Intractable Epilepsy and Cortical Dysplasia,” Epilepsia 55, no. 9 (2014): 1415–1422. 10.1111/epi.12731 25053176

[86] H. Wu , Y. Liu , H. Li , et al., “TRAK1‐Mediated Abnormality of Mitochondrial Fission Increases Seizure Susceptibility in Temporal Lobe Epilepsy,” Molecular Neurobiology 58, no. 3 (2021): 1237–1247. 10.1007/s12035-020-02175-y 33119838

[87] J. R. Vanstone , A. M. Smith , S. McBride , et al., “DNM1L‐Related Mitochondrial Fission Defect Presenting as Refractory Epilepsy,” European Journal of Human Genetics 24, no. 7 (2016): 1084–1088. 10.1038/ejhg.2015.243 PMC5070894 26604000

[88] S. Saini and S. S. Panchal , “Role of Diabetes and Its Metabolic Pathways in Epilepsy: An Insight to Various Target Approaches,” Naunyn‐Schmiedeberg's Archives of Pharmacology 398, no. 10 (2025): 13605–13623. 10.1007/s00210-025-04245-1 40347278

[89] S. Bhandary and K. Aguan , “Pyruvate Dehydrogenase Complex Deficiency and Its Relationship With Epilepsy Frequency—An Overview,” Epilepsy Research 116 (2015): 40–52. 10.1016/j.eplepsyres.2015.07.002 26354166

[90] C. Prasad , T. Rupar , and A. N. Prasad , “Pyruvate Dehydrogenase Deficiency and Epilepsy,” Brain & Development 33, no. 10 (2011): 856–865. 10.1016/j.braindev.2011.08.003 21908116

[91] R. E. Frye , “Metabolic and Mitochondrial Disorders Associated With Epilepsy in Children With Autism Spectrum Disorder,” Epilepsy & Behavior 47 (2015): 147–157. 10.1016/j.yebeh.2014.08.134 25440829

[92] A. Vetkas , J. Germann , G. Elias , et al., “Identifying the Neural Network for Neuromodulation in Epilepsy Through Connectomics and Graphs,” Brain Communications 4, no. 3 (2022): fcac092. 10.1093/braincomms/fcac092 PMC9123846 35611305

[93] J. K. Knowles , H. Xu , C. Soane , et al., “Maladaptive Myelination Promotes Generalized Epilepsy Progression,” Nature Neuroscience 25, no. 5 (2022): 596–606. 10.1038/s41593-022-01052-2 PMC9076538 35501379

[94] J. N. Gelinas and D. Khodagholy , “Interictal Network Dysfunction and Cognitive Impairment in Epilepsy,” Nature Reviews. Neuroscience 26, no. 7 (2025): 399–414. 10.1038/s41583-025-00924-3 40295879

[95] A. Friedman , D. Kaufer , and U. Heinemann , “Blood‐Brain Barrier Breakdown‐Inducing Astrocytic Transformation: Novel Targets for the Prevention of Epilepsy,” Epilepsy Research 85, no. 2–3 (2009): 142–149. 10.1016/j.eplepsyres.2009.03.005 PMC3615244 19362806

[96] A. Dadas and D. Janigro , “Breakdown of Blood Brain Barrier as a Mechanism of Post‐Traumatic Epilepsy,” Neurobiology of Disease 123 (2019): 20–26. 10.1016/j.nbd.2018.06.022 PMC6794150 30030025

[97] W. Löscher , “Epilepsy and Alterations of the Blood‐Brain Barrier: Cause or Consequence of Epileptic Seizures or Both?,” Handbook of Experimental Pharmacology 273 (2022): 331–350. 10.1007/164_2020_406 33136189

[98] A. M. S. Hartz , R. G. Rempe , E. L. B. Soldner , et al., “Cytosolic Phospholipase A2 Is a Key Regulator of Blood‐Brain Barrier Function in Epilepsy,” FASEB Journal 33, no. 12 (2019): 14281–14295. 10.1096/fj.201901369RR PMC6894050 31661303

[99] K. Leandro , J. Bicker , G. Alves , A. Falcão , and A. Fortuna , “ABC Transporters in Drug‐Resistant Epilepsy: Mechanisms of Upregulation and Therapeutic Approaches,” Pharmacological Research 144 (2019): 357–376. 10.1016/j.phrs.2019.04.031 31051235

[100] H. Song , Y. Li , J. Liu , X. Bai , L. Jiang , and T. Li , “Astrocytic Connexin 43‐Hemichannels Aggravate Seizures by Modulating Blood‐Brain Barrier Permeability in Temporal Lobe Epilepsy Mice,” Pediatric Discovery 2, no. 3 (2024): e2500. 10.1002/pdi3.2500 PMC12118272 40625459

[101] G. Yamanaka , F. Takata , Y. Kataoka , et al., “The Neuroinflammatory Role of Pericytes in Epilepsy,” Biomedicine 9, no. 7 (2021): 759. 10.3390/biomedicines9070759 PMC8301485 34209145

[102] E. A. van Vliet and N. Marchi , “Neurovascular Unit Dysfunction as a Mechanism of Seizures and Epilepsy During Aging,” Epilepsia 63, no. 6 (2022): 1297–1313. 10.1111/epi.17210 PMC9321014 35218208

[103] E. Ben‐Menachem , B. Schmitz , R. Kälviäinen , R. H. Thomas , and P. Klein , “The Burden of Chronic Drug‐Refractory Focal Onset Epilepsy: Can It Be Prevented?,” Epilepsy & Behavior 148 (2023): 109435. 10.1016/j.yebeh.2023.109435 37748414

[104] J. Liu , C. Gao , W. Chen , et al., “CRISPR/Cas9 Facilitates Investigation of Neural Circuit Disease Using Human iPSCs: Mechanism of Epilepsy Caused by an SCN1A Loss‐Of‐Function Mutation,” Translational Psychiatry 6, no. 1 (2016): e703. 10.1038/tp.2015.203 PMC5068877 26731440

[105] M. L. Lai , Y. E. Tien , Y. S. Huang , and J. D. Huang , “Studies on Pharmacokinetic Mechanism of Phenytoin Resistance in Refractory Epilepsy,” Journal of Pharmaceutical Sciences 102, no. 9 (2013): 3189–3195. 10.1002/jps.23593 23658067

[106] L. M. M. Chacón , L. G. García , K. B. García‐Ramón , et al., “Common Ictal and Interictal Perfusion Patterns: A Window Into the Epileptogenic Network and SUDEP Mechanism in Drug‐Resistant Focal Epilepsy,” Current Pharmaceutical Design 28, no. 14 (2022): 1198–1209. 10.2174/1381612828666220603125328 35658889

[107] H. Potschka , A. Fischer , W. Löscher , and H. A. Volk , “Pathophysiology of Drug‐Resistant Canine Epilepsy,” Veterinary Journal 296‐297 (2023): 105990. 10.1016/j.tvjl.2023.105990 37150317

[108] M. Pan , Q. Li , J. Song , D. Li , and R. Zhang , “Spike‐Spindle Coupling During Sleep and Its Mechanism Explanation in Childhood Focal Epilepsy,” Cognitive Neurodynamics 18, no. 5 (2024): 2145–2160. 10.1007/s11571-023-10052-2 PMC11564472 39555302

[109] L. Xiao , W. Chen , W. Guo , H. Li , R. Chen , and Q. Chen , “Exploring the Mechanism of Action of *Phyllanthus emblica* in the Treatment of Epilepsy Based on Network Pharmacology and Molecular Docking,” Medicine (Baltimore) 104, no. 7 (2025): e41414. 10.1097/MD.0000000000041414 PMC11835119 39960946

[110] S. Lu and X. Wang , “The Role and Potential Mechanism of Resveratrol in the Prevention and Control of Epilepsy,” Future Medicinal Chemistry 7, no. 15 (2015): 2005–2018. 10.4155/fmc.15.130 26505553

[111] D. Thakku Sivakumar , K. Jain , N. Alfehaid , et al., “The Purinergic P2X7 Receptor as a Target for Adjunctive Treatment for Drug‐Refractory Epilepsy,” International Journal of Molecular Sciences 25, no. 13 (2024): 6894. 10.3390/ijms25136894 PMC11241490 39000004

[112] Y. Nagahama , S. Dewar , E. Behnke , et al., “Outcome of Stereo‐Electroencephalography With Single‐Unit Recording in Drug‐Refractory Epilepsy,” Journal of Neurosurgery 139, no. 6 (2023): 1588–1597. 10.3171/2023.4.JNS222633 37243562

[113] P. P. Lenck‐Santini and S. Sakkaki , “Alterations of Neuronal Dynamics as a Mechanism for Cognitive Impairment in Epilepsy,” Current Topics in Behavioral Neurosciences 55 (2022): 65–106. 10.1007/7854_2020_193 33454922

[114] Z. Qiu , J. Guo , B. Chen , and J. Fang , “Psychosis of Epilepsy: An Update on Clinical Classification and Mechanism,” Biomolecules 15, no. 1 (2025): 56. 10.3390/biom15010056 PMC11762389 39858450

[115] L. D. Godoy , T. Prizon , M. T. Rossignoli , J. P. Leite , and J. L. Liberato , “Parvalbumin Role in Epilepsy and Psychiatric Comorbidities: From Mechanism to Intervention,” Frontiers in Integrative Neuroscience 16 (2022): 765324. 10.3389/fnint.2022.765324 PMC8891758 35250498

[116] H. Zhao , G. Lin , M. Shi , et al., “The Mechanism of Neurogenic Pulmonary Edema in Epilepsy,” Journal of Physiological Sciences 64, no. 1 (2014): 65–72. 10.1007/s12576-013-0291-6 PMC10717646 24142459

[117] X. Ji , Q. Pei , J. Zhang , et al., “Single‐Cell Sequencing Combined With Machine Learning Reveals the Mechanism of Interaction Between Epilepsy and Stress Cardiomyopathy,” Frontiers in Immunology 14 (2023): 1078731. 10.3389/fimmu.2023.1078731 PMC9911815 36776884

[118] L. Sun , J. Gilligan , C. Staber , et al., “A Knock‐In Model of Human Epilepsy in Drosophila Reveals a Novel Cellular Mechanism Associated With Heat‐Induced Seizure,” Journal of Neuroscience 32, no. 41 (2012): 14145–14155. 10.1523/JNEUROSCI.2932-12.2012 PMC3482260 23055484

[119] A. Zahra , R. Liu , J. Wang , and J. Wu , “Identifying the Mechanism of Action of the Kv7 Channel Opener, Retigabine in the Treatment of Epilepsy,” Neurological Sciences 44, no. 11 (2023): 3819–3825. 10.1007/s10072-023-06955-x 37442907

[120] R. Liu , L. Sun , Y. Wang , Q. Wang , and J. Wu , “New Use for an Old Drug: Quinidine in KCNT1‐Related Epilepsy Therapy,” Neurological Sciences 44, no. 4 (2023): 1201–1206. 10.1007/s10072-022-06521-x 36437393

[121] Y. Ru , G. An , Z. Wei , and H. Chen , “Epilepsy Detection Based on Multi‐Head Self‐Attention Mechanism,” PLoS One 19, no. 6 (2024): e0305166. 10.1371/journal.pone.0305166 PMC11166279 38861543

[122] E. H. Middlebrooks , S. S. Grewal , M. Stead , B. N. Lundstrom , G. A. Worrell , and J. J. Van Gompel , “Differences in Functional Connectivity Profiles as a Predictor of Response to Anterior Thalamic Nucleus Deep Brain Stimulation for Epilepsy: A Hypothesis for the Mechanism of Action and a Potential Biomarker for Outcomes,” Neurosurgical Focus 45, no. 2 (2018): E7. 10.3171/2018.5.FOCUS18151 30064322

[123] B. Hu , Z. Wang , M. Xu , L. Zhu , and D. Wang , “The Inhibition Mechanism of Epilepsy Disease in a Computational Model,” Technology and Health Care 30, no. S1 (2022): 155–162. 10.3233/THC-228015 PMC9028747 35124593

[124] B. Hu , Z. Wang , M. Xu , L. Zhu , and D. Wang , “The Therapeutic Mechanism of Epilepsy Seizures in Different Target Areas: Research on a Theoretical Model,” Technology and Health Care 29, no. S1 (2021): 455–461. 10.3233/THC-218043 PMC8150464 33682782

[125] B. Li , L. Wang , and B. Tu , “Editorial: The Mechanism of Sudden Unexpected Death in Epilepsy and the Specific Forensic Diagnostic Indicators in Sudden Death With a Negative Autopsy,” Frontiers in Neurology 14 (2023): 1265787. 10.3389/fneur.2023.1265787 PMC10471961 37662037

[126] W. Shao , L. Liu , J. Gu , et al., “Spotlight on Mechanism of Sudden Unexpected Death in Epilepsy in Dravet Syndrome,” Translational Psychiatry 15, no. 1 (2025): 84. 10.1038/s41398-025-03304-8 PMC11914262 40097380

[127] X. Sun , Y. Lv , and J. Lin , “The Mechanism of Sudden Unexpected Death in Epilepsy: A Mini Review,” Frontiers in Neurology 14 (2023): 1137182. 10.3389/fneur.2023.1137182 PMC9939452 36815002

[128] R. Y. Jiang , R. T. Varughese , and S. V. Kothare , “Sudden Unexpected Death in Epilepsy: A Narrative Review of Mechanism, Risks, and Prevention,” Journal of Clinical Medicine 14, no. 10 (2025): 3329. 10.3390/jcm14103329 PMC12112541 40429323

[129] M. Biet , N. Morin , M. Lessard‐Beaudoin , et al., “Prolongation of Action Potential Duration and QT Interval During Epilepsy Linked to Increased Contribution of Neuronal Sodium Channels to Cardiac Late Na+ Current: Potential Mechanism for Sudden Death in Epilepsy,” Circulation. Arrhythmia and Electrophysiology 8, no. 4 (2015): 912–920. 10.1161/CIRCEP.114.002693 26067667

[130] E. Glasscock , J. W. Yoo , T. T. Chen , T. L. Klassen , and J. L. Noebels , “Kv1.1 Potassium Channel Deficiency Reveals Brain‐Driven Cardiac Dysfunction as a Candidate Mechanism for Sudden Unexplained Death in Epilepsy,” Journal of Neuroscience 30, no. 15 (2010): 5167–5175. 10.1523/JNEUROSCI.5591-09.2010 PMC2874941 20392939

[131] M. Dupuis , K. van Rijckevorsel , F. Evrard , N. Dubuisson , F. Dupuis , and P. Van Robays , “Takotsubo Syndrome (TKS): A Possible Mechanism of Sudden Unexplained Death in Epilepsy (SUDEP),” Seizure 21, no. 1 (2012): 51–54. 10.1016/j.seizure.2011.09.006 21996459

[132] R. B. Budde , M. A. Arafat , D. J. Pederson , T. A. Lovick , J. G. R. Jefferys , and P. P. Irazoqui , “Acid Reflux Induced Laryngospasm as a Potential Mechanism of Sudden Death in Epilepsy,” Epilepsy Research 148 (2018): 23–31. 10.1016/j.eplepsyres.2018.10.003 PMC6276113 30336367

[133] M. Lucchesi , J. B. Silverman , K. Sundaram , R. Kollmar , and M. Stewart , “Proposed Mechanism‐Based Risk Stratification and Algorithm to Prevent Sudden Death in Epilepsy,” Frontiers in Neurology 11 (2020): 618859. 10.3389/fneur.2020.618859 PMC7868441 33569036

[134] R. Mandal , R. Budde , G. L. Lawlor , and P. Irazoqui , “Utilizing Multimodal Imaging to Visualize Potential Mechanism for Sudden Death in Epilepsy,” Epilepsy & Behavior 122 (2021): 108124. 10.1016/j.yebeh.2021.108124 PMC8429091 34237676

[135] M. J. Gunthorpe , C. H. Large , and R. Sankar , “The Mechanism of Action of Retigabine (Ezogabine), a First‐In‐Class K+ Channel Opener for the Treatment of Epilepsy,” Epilepsia 53, no. 3 (2012): 412–424. 10.1111/j.1528-1167.2011.03365.x 22220513

[136] P. Dong , Y. Zhang , A. S. Hunanyan , M. A. Mikati , J. Cui , and H. Yang , “Neuronal Mechanism of a BK Channelopathy in Absence Epilepsy and Dyskinesia,” Proceedings of the National Academy of Sciences of the United States of America 119, no. 12 (2022): e2200140119. 10.1073/pnas.2200140119 PMC8944272 35286197

[137] J. Clatot , C. B. Currin , Q. Liang , et al., “A Structurally Precise Mechanism Links an Epilepsy‐Associated KCNC2 Potassium Channel Mutation to Interneuron Dysfunction,” Proceedings of the National Academy of Sciences of the United States of America 121, no. 3 (2024): e2307776121. 10.1073/pnas.2307776121 PMC10801864 38194456

[138] V. Golub and D. S. Reddy , “Cannabidiol Therapy for Refractory Epilepsy and Seizure Disorders,” Advances in Experimental Medicine and Biology 1264 (2021): 93–110. 10.1007/978-3-030-57369-0_7 33332006

[139] F. Nikbakht , P. Hashemi , S. Vazifekhah , and J. F. Babaei , “Investigating the Mechanism of Antiepileptogenic Effect of Apigenin in Kainate Temporal Lobe Epilepsy: Possible Role of mTOR,” Experimental Brain Research 241, no. 3 (2023): 753–763. 10.1007/s00221-023-06557-1 36719442

[140] A. M. D'Gama and A. Poduri , “Precision Therapy for Epilepsy Related to Brain Malformations,” Neurotherapeutics 18, no. 3 (2021): 1548–1563. 10.1007/s13311-021-01122-6 PMC8608994 34608615

[141] M. J. Benson , N. K. Thomas , S. Talwar , et al., “A Novel Anticonvulsant Mechanism via Inhibition of Complement Receptor C5ar1 in Murine Epilepsy Models,” Neurobiology of Disease 76 (2015): 87–97. 10.1016/j.nbd.2015.02.004 25681535

[142] P. Therajaran , J. A. Hamilton , T. J. O'Brien , N. C. Jones , and I. Ali , “Microglial Polarization in Posttraumatic Epilepsy: Potential Mechanism and Treatment Opportunity,” Epilepsia 61, no. 2 (2020): 203–215. 10.1111/epi.16424 31943156

[143] J. W. Miller , “Inflammation as a Target for Epilepsy Therapy: The Case of Natalizumab,” Neurology 97, no. 18 (2021): 845–846. 10.1212/WNL.0000000000012768 34521688

[144] J. Wang , W. Wu , J. Wan , et al., “Preliminary Study on the Mechanism of SAHA in the Treatment of Refractory Epilepsy Induced by GABRG2(F343L) Mutation,” Biochemical Pharmacology 227 (2024): 116449. 10.1016/j.bcp.2024.116449 39053637

[145] A. Parras , L. de Diego‐Garcia , M. Alves , et al., “Polyadenylation of mRNA as a Novel Regulatory Mechanism of Gene Expression in Temporal Lobe Epilepsy,” Brain 143, no. 7 (2020): 2139–2153. 10.1093/brain/awaa168 32594159

[146] J. Zhao , X. Qin , L. Yang , et al., “Application of TCM Network Pharmacology and Experimental Verification to Explore the Mechanism of Kaempferol Against Epilepsy,” Brain Research Bulletin 220 (2025): 111150. 10.1016/j.brainresbull.2024.111150 39608614

[147] W. Wang , Y. Zhang , Y. Yang , and L. Gu , “Network Pharmacology and Molecular Docking to Explore the Mechanism of Kangxian Decoction for Epilepsy,” Evidence‐Based Complementary and Alternative Medicine 2022 (2022): 3333878. 10.1155/2022/3333878 PMC9525756 36193133

[148] H. Singh , A. Raja , A. Chauhan , et al., “Unlocking the Therapeutic Potential: Sitagliptin's Multifaceted Approach in Drug‐Resistant Epilepsy Through a Novel Mechanism Inhibiting Protein Kinase C‐γ and a Long‐Term Potentiation Pathway,” ACS Pharmacology & Translational Science 7, no. 6 (2024): 1856–1863. 10.1021/acsptsci.4c00073 PMC11184596 38898950

[149] S. A. Litvinova , T. A. Voroninа , L. N. Nerobkova , I. S. Kutepova , G. G. Avakyan , and G. N. Avakyan , “Levetiracetam Effect and Electrophysiological Mechanism of Action in Rats With Cobalt‐Induced Chronic Epilepsy,” European Journal of Pharmacology 854 (2019): 380–386. 10.1016/j.ejphar.2019.04.041 31034820

[150] H. Kikuyama , T. Hanaoka , T. Kanazawa , et al., “The Mechanism of Anti‐Epileptogenesis by Levetiracetam Treatment Is Similar to the Spontaneous Recovery of Idiopathic Generalized Epilepsy During Adolescence,” Psychiatry Investigation 14, no. 6 (2017): 844–850. 10.4306/pi.2017.14.6.844 PMC5714728 29209390

[151] R. Fisher , V. Salanova , T. Witt , et al., “Electrical Stimulation of the Anterior Nucleus of Thalamus for Treatment of Refractory Epilepsy,” Epilepsia 51, no. 5 (2010): 899–908. 10.1111/j.1528-1167.2010.02536.x 20331461

[152] V. Salanova , T. Witt , R. Worth , et al., “Long‐Term Efficacy and Safety of Thalamic Stimulation for Drug‐Resistant Partial Epilepsy,” Neurology 84, no. 10 (2015): 1017–1025. 10.1212/WNL.0000000000001334 PMC4352097 25663221

[153] M. Abdennadher , P. Rohatgi , and A. Saxena , “Vagus Nerve Stimulation Therapy in Epilepsy: An Overview of Technical and Surgical Method, Patient Selection, and Treatment Outcomes,” Brain Sciences 14, no. 7 (2024): 675. 10.3390/brainsci14070675 PMC11275221 39061416

[154] J. W. Wheless , J. S. Raskin , A. L. Fine , et al., “Expert Opinion on Use of Vagus Nerve Stimulation Therapy in the Management of Pediatric Epilepsy: A Delphi Consensus Study,” Seizure 123 (2024): 97–103. 10.1016/j.seizure.2024.10.013 39536380

[155] M. J. Morrell , “Responsive Cortical Stimulation for the Treatment of Medically Intractable Partial Epilepsy,” Neurology 77, no. 13 (2011): 1295–1304. 10.1212/WNL.0b013e3182302056 21917777

[156] D. R. Nair , K. D. Laxer , P. B. Weber , et al., “Nine‐Year Prospective Efficacy and Safety of Brain‐Responsive Neurostimulation for Focal Epilepsy,” Neurology 95, no. 9 (2020): e1244–e1256. 10.1212/WNL.0000000000010154 PMC7538230 32690786

[157] X. Guo , C. Wang , and J. Guo , “The Effect of Mozart's K.448 on Epilepsy: A Systematic Literature Review and Supplementary Research on Music Mechanism,” Epilepsy & Behavior 163 (2025): 110108. 10.1016/j.yebeh.2024.110108 39637732

[158] J. J. Van Gompel , D. B. Burkholder , J. J. Parker , et al., “Laser Interstitial Thermal Therapy for Epilepsy,” Neurosurgery Clinics of North America 34, no. 2 (2023): 247–257. 10.1016/j.nec.2022.11.005 36906331

[159] P. Landazuri , J. J. Cheng , E. Leuthardt , et al., “Interstitial Thermal Therapy in Mesial Temporal Lobe Epilepsy,” JAMA Neurology 82, no. 9 (2025): 915–924. 10.1001/jamaneurol.2025.1897 PMC12235534 40622685

[160] A. Ko , H. E. Kwon , and H. D. Kim , “Updates on the Ketogenic Diet Therapy for Pediatric Epilepsy,” Biomed Journal 45, no. 1 (2022): 19–26. 10.1016/j.bj.2021.11.003 PMC9133260 34808422

[161] G. R. Lum , S. M. Ha , C. A. Olson , et al., “Ketogenic Diet Therapy for Pediatric Epilepsy Is Associated With Alterations in the Human Gut Microbiome That Confer Seizure Resistance in Mice,” Cell Reports 42, no. 12 (2023): 113521. 10.1016/j.celrep.2023.113521 PMC10769314 38070135

[162] X. Tong , Q. Wang , J. Yang , et al., “Optimizing Ketogenic Diet Therapy for Childhood Epilepsy: Identifying Key Factors for Seizure Control and Psychomotor Enhancement,” Epilepsia 65, no. 10 (2024): 2959–2972. 10.1111/epi.18098 39190400

[163] J. Zhao , J. Tang , and X. Shi , “Mechanism of miR‐4465 Targeting PTEN‐Mediated Autophagy of Astrocytes in Epilepsy,” Journal of Medical Biochemistry 44, no. 6 (2025): 1347–1356. 10.5937/jomb0-52626 PMC12497466 41054573

